# Serious adverse events reported in placebo randomised controlled trials of oral naltrexone: a systematic review and meta-analysis

**DOI:** 10.1186/s12916-018-1242-0

**Published:** 2019-01-15

**Authors:** Monica Bolton, Alex Hodkinson, Shivani Boda, Alan Mould, Maria Panagioti, Sarah Rhodes, Lisa Riste, Harm van Marwijk

**Affiliations:** 1 School of Health Sciences, Faculty of Biology, Medicine and Health, University of Manchester, Manchester, M13 9PL UK; 2 Centre for Primary Care, Division of Population Health, Health Services Research & Primary Care, Williamson Building, Oxford Road, Manchester, M13 9PL UK; 3 School of Medical Sciences, Faculty of Biology, Medicine and Health, University of Manchester, Manchester, M13 9PL UK; 4 Centre for Biostatistics, Division of Population Health, Health Services Research and Primary Care, School of Health Sciences, Faculty of Biology, Medicine and Health, University of Manchester, Manchester, M13 9PL UK; 5 Division of Population Health, Health Services Research and Primary Care, School of Health Sciences, Faculty of Biology, Medicine and Health, University of Manchester, Manchester, M13 9PL UK; 6 Brighton and Sussex Medical School, Watson Building, University of Brighton, Brighton, BN1 9PH UK

**Keywords:** Naltrexone, Serious adverse events, Systematic review, Low dose naltrexone, LDN, alcohol use disorder

## Abstract

**Background:**

Naltrexone is an opioid antagonist used in many different conditions, both licensed and unlicensed. It is used at widely varying doses from 3 to 250 mg. The aim of this review was to extensively evaluate the safety of oral naltrexone by examining the risk of serious adverse events and adverse events in randomised controlled trials of naltrexone compared to placebo.

**Methods:**

A systematic search of the Cochrane Central Register of Controlled Trials, MEDLINE, Embase, other databases and clinical trials registries was undertaken up to May 2018. Parallel placebo-controlled randomised controlled trials longer than 4 weeks published after 1 January 2001 of oral naltrexone at any dose were selected. Any condition or age group was included, excluding only studies in opioid or ex-opioid users owing to possible opioid/opioid antagonist interactions. The systematic review used the guidance of the Cochrane Handbook and Preferred Reporting Items for Systematic Reviews and Meta-analyses harms checklist throughout. Numerical data were independently extracted by two people and cross-checked. Risk of bias was assessed with the Cochrane risk-of-bias tool. Meta-analyses were performed in R using random effects models throughout.

**Results:**

Eighty-nine randomised controlled trials with 11,194 participants were found, studying alcohol use disorders (n = 38), various psychiatric disorders (n = 13), impulse control disorders (n = 9), other addictions including smoking (n = 18), obesity or eating disorders (n = 6), Crohn’s disease (n = 2), fibromyalgia (n = 1) and cancers (n = 2). Twenty-six studies (4,960 participants) recorded serious adverse events occurring by arm of study. There was no evidence of increased risk of serious adverse events for naltrexone compared to placebo (risk ratio 0.84, 95% confidence interval 0.66–1.06). Sensitivity analyses pooling risk differences supported this conclusion (risk difference −0.01, 95% confidence interval −0.02–0.00) and subgroup analyses showed that results were consistent across different doses and disease groups. Secondary analysis revealed only six marginally significant adverse events for naltrexone compared to placebo, which were of mild severity.

**Conclusions:**

Naltrexone does not appear to increase the risk of serious adverse events over placebo. These findings confirm the safety of oral naltrexone when used in licensed indications and encourage investments to undertake efficacy studies in unlicensed indications.

**Trial registration:**

PROSPERO 2017 CRD42017054421.

**Electronic supplementary material:**

The online version of this article (10.1186/s12916-018-1242-0) contains supplementary material, which is available to authorized users.

## Background

Naltrexone is a pure opioid antagonist with activity at multiple opioid and non-opioid human receptors. Its licensed uses are as an aid to prevent relapse in alcohol use disorders (AUDs) and opioid addiction after withdrawal, and in the combination tablet naltrexone–bupropion for obesity [1]. These conditions are all major global health problems, with rising rates of disability and death occurring in many countries [2, 3]. Despite concern about the impact of these diseases and the need for treatment, naltrexone is currently under-utilised across most countries, particularly for AUDs [4–6].

At normal or higher doses (≥50 mg), naltrexone is also used off-label for several addictions and impulse control disorders that currently have no licensed drug treatments, such as amphetamine and cocaine addiction [7, 8], impulse control disorders [9–11], eating disorders [12] and autism spectrum disorders [13].

Following experimental findings that low doses of naltrexone result in tumour growth suppression [14] and immune modulation [15], it is increasingly used at doses of around 4.5 mg. This is known as low dose naltrexone (LDN). Small-scale clinical trials of LDN have been conducted in, for example, Crohn’s disease, multiple sclerosis, fibromyalgia and HIV infection, where the evidence has shown efficacy and/or low toxicity [16–19]. Other conditions for which LDN is used, such as chronic fatigue syndrome (also known as myalgic encephalomyelitis), complex regional pain syndrome and auto-immune disorders, are still awaiting randomised clinical trials (RCTs) [20–22]. LDN is now licensed as an adjunct in HIV infection for over-the-counter sales in Kenya and Nigeria [23]. In Norway, it has been associated with a reduction in prescriptions for more conventional treatments in some conditions [24, 25]. In the UK, around 1,400 NHS prescriptions for LDN are issued per year ([26]; personal communication from D. Steinke, October 2016: LDN usage in CPRD), while over 12,000 people have received a private prescription in the last 10 years (personal communication from S. Dickson, October 2017: LDN private prescriptions dispensed from Dicksons Chemist Glasgow in past 10 years).

### Known safety issues for naltrexone

Naltrexone is contra-indicated in those currently using opioids due to the possibility of serious adverse events (SAEs) of either over-rapid opioid withdrawal or overdose of opioids, which can be life-threatening [1, 27]. These SAEs are of a different nature from those occurring in non-opioid users.

Concerns about naltrexone causing liver toxicity originated from several high-dose studies (up to 300 mg) in the 1980s [28]. Because of these results, the US Food and Drug Administration (FDA) initially required a “black-box warning” about hepatotoxicity in the package insert for naltrexone; the FDA specifies such warnings to call attention to serious or life-threatening risks. However, because there are no known cases of hepatic failure due to naltrexone [29, 30], the warning was eventually removed in 2013 [27]. The British National Formulary cautions avoidance in cases with acute hepatitis, hepatic failure or severe impairment, and in severe renal impairment. Known side effects include nausea, vomiting, abdominal pain, decreased appetite, dizziness, lethargy, headaches and sleep disorders [1, 29].

### Drug safety in clinical trials

The quality of recording and reporting of harms in clinical trials has historically been less rigorous than that of efficacy [31, 32]. Progress has been aided by the introduction of standard definitions for adverse events (Box 1); the requirement to keep detailed records of adverse events (AEs) in clinical trials, introduced in 2001; the International Committee of Medical Journal Editors’ endorsement of the reporting standards suggested in the Consolidated Standards of Reporting Trials (CONSORT) extension for harms published in 2004 [33], and the Preferred Reporting Items for Systematic Reviews and Meta-analyses (PRISMA) harms checklist published in 2016 [34]; and the requirement to record outcomes, including AEs and SAEs, for RCTs registered on clinical trials registries since 2014 in the European Union (EU) [35] and 2017 in the USA [36]. An evidence synthesis of harms (SAEs and AEs) would help to yield a more accurate safety profile of naltrexone.

### Why is it important to do this review?

There have been several descriptive, non-systematic safety reviews of naltrexone recently [37, 38], but none to date have concentrated on AEs and SAEs in clinical trials of naltrexone. Including studies from a wide range of conditions and concentrating only on a specific adverse outcome that has a regulatory definition should enable a large quantity of high-quality harms data to be collected. People with addictions are reluctant to take, and therapists to prescribe, one drug to overcome addiction to another drug, including alcohol [39, 40], and practitioners remain concerned about the risk for liver toxicity with naltrexone [41–43]. Hence, evidence about the safety of naltrexone is needed. Patients taking naltrexone and LDN may do so for prolonged periods of time; therefore, establishing the longer-term safety of naltrexone is particularly important. It may only be possible to discover increased or decreased rates of some SAEs, particularly cardiovascular or cerebrovascular events or cancers in meta-analyses, owing to their generally low background rates [44].

The primary aim of this review was to examine SAEs occurring in clinical trials of oral naltrexone, given for any condition apart from opioid or ex-opioid use, compared to placebo. Our focus on SAEs accords with the recent emphasis on understanding and preventing enduring or permanent patient harm (rather than examining every AE), as highlighted, for example, in the Dalton review of duty of candour [45]. Further aims were to investigate possible confounders of risk of SAEs for naltrexone by subgroup analyses of disease group, dosage and length of study; to examine specific SAEs (deaths, cardiovascular or cerebrovascular events and cancers); and to examine withdrawals and withdrawals due to AEs in the same clinical trials. A secondary aim was to examine AEs for naltrexone compared to placebo.

## Methods

The review followed the Cochrane Handbook for guidance throughout [46] and the PRISMA harms extension [34]. The protocol was registered on the PROSPERO website in January 2017, registration number CRD42017054421. This can be accessed at https://www.crd.york.ac.uk/PROSPERO/display_record.asp?ID=CRD42017054421.

### Selection criteria

Any parallel-designed RCT longer than 4 weeks, in participants of any age and for any condition, in which oral naltrexone was compared to placebo was included. Studies in which opioid or ex-opioid use was specified in the protocol were excluded owing to the possibility of opioid/opioid antagonist interactions occurring. Only studies published after 1 January 2001 were included, owing to the widespread introduction of regulations requiring the recording of AEs and reporting of SAEs in RCTs from that year [47].

### Outcomes

The primary outcome measure was the number of participants with an SAE recorded in the naltrexone arm compared to the placebo arm. The investigator’s judgement as to whether an SAE had occurred and any causality was followed, as suggested by the International Conference on Harmonisation (ICH) (Box 1). Where no definition was given, the definition(s) from the detailed guidance CT-3 for the EU [48] and by the FDA for the USA [49] as summarised in Box 1 was used to support our judgement. The secondary outcome was the type of AEs reported in either treatment arm.

### Search methods for identifying studies

The following electronic databases were searched: Cochrane Central Register of Controlled Trials (CENTRAL), PubMed MEDLINE, EMBASE (via OVID), Web of Science Core Collection, PsycINFO (via OVID) and International Pharmaceutical Abstracts via OVID (Additional file 1). There were no language restrictions. No terms for AE or side effects were included to avoid over-restrictive selection of studies with the potential risk of outcome reporting bias [51–53]. The final date of searches was May 2018.

Further sources were relevant systematic reviews containing clinical trials of naltrexone, and journal articles being assessed for inclusion in this review. The World Health Organization International Clinical Trials Registry, the US clinical trials registry, clinicaltrials.gov and the European Union Clinical Trials Registry EudraCT were searched using the word “naltrexone”. These are good sources of unpublished but completed clinical trials [36]. Where a study appeared unpublished, the lead investigator was contacted to confirm this was so. Ongoing studies were recorded, to enable future updating of this systematic review. Grey literature was included in the review from clinical trial registries, conference abstracts listed within CENTRAL, regulatory submissions to the US FDA for drug licences and unpublished studies located from previous systematic reviews.

### Data collection and management

All screening and data extraction were undertaken by two researchers independently (MB and SB for screening and MB and AM for data extraction), and results were compared to draw up a final list. Any differences were resolved by discussion, with occasional input from a third reviewer (HvM, MP, SR or LR). Initial screening eliminated studies using the title and abstract, with full papers examined to select the final included studies. All searches were downloaded to Endnote referencing software, where duplicates of papers were removed, and multiple papers linked to the same study identified. The numbers found at each stage and reasons for decisions were recorded.

Data were recorded on data extraction forms. Quantitative data for the primary and secondary outcomes, enrolment numbers and withdrawals (numbers and reasons), SAEs (both number of participants with an SAE and total number of SAEs, and descriptions) and AEs (total numbers per Medical Dictionary for Regulatory Activities (MedDRA) preferred term) were extracted onto an Excel spreadsheet. Website appendices, subsidiary studies and any published protocols were examined for relevant information. Results on clinicaltrials.gov and on EudraCT were cross-checked with the data available in the study report.

### Quality assessment

The Cochrane risk-of-bias tool [54] was adapted for the outcome measures in this review, highlighting eight areas of trial conduct and reporting. The CONSORT extension for harms [33] was used to inform the choice of criteria. The areas chosen were:

- Random sequence generation (selection bias)
- Allocation concealment (selection bias)
- Blinding of participants and personnel to randomisation (performance bias)
- Blinding of outcome assessment (detection bias)
- Adequate outcome data reporting (attrition bias)
- Adequate collection of AEs and SAEs (attrition bias)
- Adequate reporting of SAEs (reporting bias)
- Other bias (e.g. commercial sponsorship, placebo run-in periods)

A risk of bias table was drawn up which included the comments drawn directly from the papers, followed by a judgement for each study. Judgements were made by MB and all decisions reviewed by SR, with occasional discussions with a third reviewer in order to reach consensus. The results were used to identify studies at low risk of bias in all eight categories (low risk studies), the remaining studies having at least one category judged not low risk.

### Measures of treatment effect

A corresponding meta-analysis was performed using data extracted from journal publications and other sources (clinicaltrials.gov and data supplied by authors) wherever relevant. The pooled risk ratio (RR) was compared across trials reporting SAEs in the naltrexone arm compared to the placebo arm for events recorded during active treatment (naltrexone or placebo). Because participants may have multiple identical SAEs in one clinical trial, or more than one SAE which could be related, the RR was analysed per participant rather than per event. A sensitivity analysis of risk difference (RD) was performed because it uses data from all studies including those with no events in either arm. Meta-analyses for the RR of individual MedDRA preferred-term AEs, withdrawals, withdrawals due to AEs and deaths were also performed. Associated 95% confidence intervals were recorded throughout.

### Data synthesis and assessment of heterogeneity

Although there was clinical heterogeneity among studies, meta-analysis was appropriate because the comparator and outcome measure was the same for all studies and the direction of effect was likely to be similar [55]. The programme R was used for all meta-analyses (R Foundation for Statistical Computing, Vienna, Austria). Studies with events in one arm only were included by applying the continuity correction of adding 0.5 to all cells of a 2 × 2 table of results for each study [56, 57]. Double zero studies (i.e. studies that report zero events in each treatment arm) were excluded from the analysis, as recommended in the Cochrane Handbook. Data were analysed on an intention-to-treat basis. Clinical heterogeneity was acknowledged by using random effects models in all analyses. Statistical heterogeneity was examined with the *I*^2^ statistic. Values of less than 25% represent low heterogeneity, and above 75% represent high heterogeneity [55]. A univariate and multivariate meta-regression was used to explore further causes of heterogeneity involving covariates, including age, gender, year of publication, length of trial and quality of study (i.e. low or high risk of bias).

### Studies with multiple treatment groups

Studies trialling multiple drugs or therapies (e.g. drug plus naltrexone compared to drug, or a four-arm factorial design) were included when there was a suitable placebo arm for comparison with naltrexone. Studies with a fixed combination of naltrexone and another drug in which the comparator was a single placebo were not included. This excluded the combination tablet of slow-release naltrexone–bupropion. In studies with multiple naltrexone arms and only one placebo arm (e.g. if different dosages of naltrexone were trialled), data from the placebo arm were divided to match the naltrexone arms by the proportions of participants recruited to each naltrexone arm. This avoided any double counting of the placebo arm. In trials with multiple psychotherapeutic interventions in different arms, the results of these could be combined, as long as the same interventions were in the placebo arms.

### Missing data

If data were missing or ambiguous (e.g. if it was not clear from a paper if SAEs occurred or if there were discrepancies in data between the paper and the website clinicaltrials.gov), lead authors were contacted for further information. Records were kept of all such correspondence, including where this resulted in changes to the data. In studies lacking specific comments on SAEs, judgement was sometimes necessary to determine the presence or absence of SAEs, depending on the extent of information provided about AEs. Reasons for decisions were recorded, quoting the relevant text or table from the study. All studies involving judgements on data were judged unclear for risk of reporting bias.

### Subgroup and sensitivity analyses

Subgroup analyses of disease or condition and dose were defined a priori with a rationale for why such differences in rates of SAEs may exist [55]. Additionally, length of study was added as a post hoc analysis owing to its potential modifying effect on rates of SAEs. Sensitivity analyses including only studies at low risk of bias in all categories explored the robustness of findings to risks of bias [55]. Other sensitivity analyses were explored following results of data analysis to test the robustness of the findings.

### Assessment of reporting biases

This review attempted to reduce publication bias by using wide-ranging search strategies, by including publications that were not in English, and by looking for unreported clinical trials on clinical trials registries. Reporting bias was assessed visually for each meta-analysis using funnel plots and the relevant statistical analyses.

## Results

### Trial flow: flow diagram and numbers

**Fig 1:**
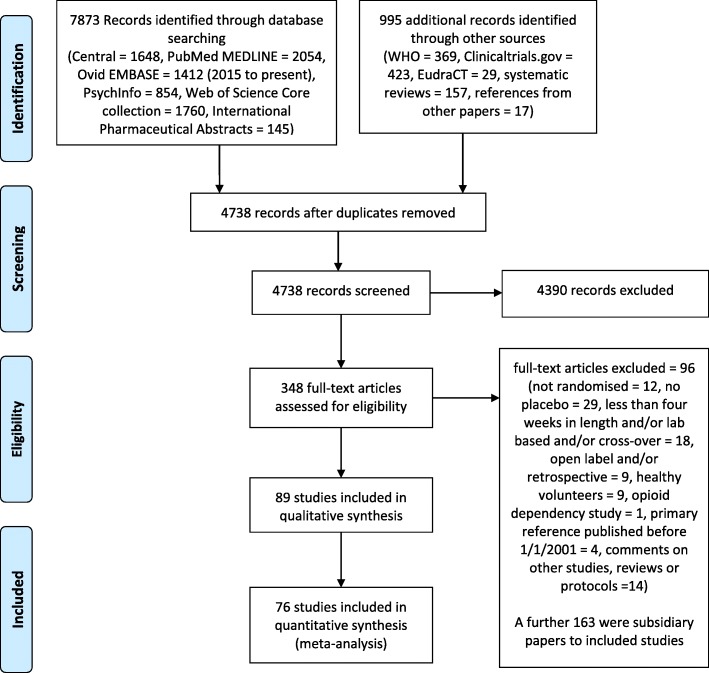
PRISMA 2009 flow diagram. WHO World Health Organization; EudraCT European Clinical Trials Database

The electronic searches identified 7873 citations, and a further 995 records identified from clinical trials websites (821), systematic reviews (157) and references in other papers (17). Deleting duplicate references reduced this to 4738 records, of which 4390 were excluded on the basis of examining the abstracts. Full-text articles were obtained for 348 citations. From these, 96 citations were excluded and 163 were subsidiary papers. Thus, 89 primary studies were identified (Additional file 2). The numbers identified at each stage through from initial searching to quantitative analyses, and the reasons for excluding studies, are given in a PRISMA 2009 flow diagram (Fig. 1) [58].

### Characteristics of included studies

Eighty-nine studies (11,194 participants) were found that fulfilled the review criteria, including publication after 1 January 2001. Three studies were excluded because they only gave total participant numbers, leaving 86 studies (10,957 participants) from which data could potentially be extracted for analysis. Table 1 summarises the characteristics of included studies by broad categories, while Additional file 3: Table S1 provides the details of each study. The target dose of naltrexone varied from 3 mg to 250 mg. The most frequent conditions were AUDs (36 studies). In a further 21 studies, including studies of HIV infection, psychiatric disorders, addictions and smoking, participants had a dual diagnosis including AUDs. Other studies were of various psychiatric disorders, impulse control disorders, other addictions, obesity, Crohn’s disease, fibromyalgia and cancers. The patient groups in many of the studies tended to have complex problems, multi-morbidity, present or past multiple addictions or illnesses and to be taking multiple drugs as well as the trial drugs.

**Table 1:** Summary of characteristics of included studies

Category	Characteristics of participants in study	Number of studies
Disease or condition	AUD	38
	Drug addiction or smoking ± AUD	18
	Psychiatric disorders ± AUD	13
	Impulse control disorders	9
	Obesity or eating disorders	6
	Inflammatory disorders	3
	malignancies	2
Target dose of naltrexone^a^ (mg)	≤4.5	5
	16–49	7
	50	61
	100	12
	>100	8
Mean age where given^b^ (years)	10 to <20	2
	20 to <30	2
	30 to <40	11
	40 to <50	62
	50 to <60	3
	≥60	2
Length of study^c^ (weeks)	4–7	5
	8–11	17
	12–15	42
	16–25	18
	26–52	6

*AUD* alcohol use disorder

^a^3 studies were multi-arm dose-finding studies

^b^82 studies

^c^88 studies

### Results of the quality assessment

The results of the Cochrane risk of bias assessments for all studies are summarised in Additional file 4: Table S2. Twelve studies were judged to have a low risk of bias in all eight categories. These studies enrolled a total of 2,540 participants (28%). Eighteen studies (20%) were low risk for six or seven of the categories, and 14 studies (16%) were low risk in two or fewer categories.

### Prevalence and nature of serious adverse events

In events ascribed to a particular study arm, naltrexone or placebo, a total of 315 SAEs were recorded among 260 participants. The number of participants having at least one SAE was 119 in the naltrexone arms and 141 in the placebo arms. Among the 315 SAEs, nine deaths were reported, three in the naltrexone arms and six in the placebo arms. Although examining the nature and causality of SAEs was beyond the scope of this study, wherever such data were provided, they were extracted. Our descriptive review of these limited data suggested that there were no differences between the two treatment arms in terms of the nature of SAEs. Among the included studies, AEs were reported across 20 independent comparisons. A total of 7,017 AEs (involving 188 MedDRA preferred-term events) were identified: 3,938 in the naltrexone arm and 3,079 in the placebo arm (Additional file 5: Table S3). All AEs were reported as being mild-moderate in nature.

### Statistical tests and results

#### Serious adverse events

**Fig 2:**
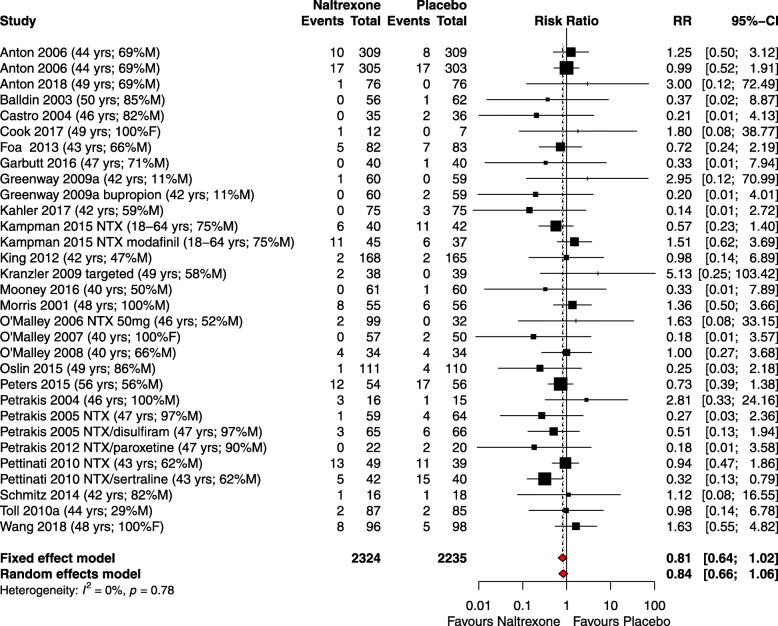
Forest plot of risk ratio (RR) of serious adverse events in RCTs of naltrexone vs placebo. Data in parentheses show the mean or range of participants’ age and the percentage of male or female participants. Double zero studies (i.e. those which reported zero events in each treatment group) were excluded from the meta-analysis. This also applies for all the subgroup analyses (for dose, disease and time)

There was no evidence of any difference between naltrexone and placebo in the meta-analysis of RR of SAEs. A total of 31 comparisons from the 26 studies recording the number of SAEs by study arm were analysed. The pooled RR for the number of participants experiencing at least one SAE for naltrexone compared to placebo was not statistically significant (RR 0.84, 95% CI 0.66–1.06). Tests for heterogeneity showed low statistical heterogeneity (*I*^2^ = 0%). The forest plot for this result is shown in Fig. 2. The pooled RD for the number of participants experiencing at least one SAE for naltrexone compared to placebo was non-significant (RD −0.01, 95% CI −0.02–0.00). Heterogeneity was low (*I*^2^ = 7%). The forest plot for RR of death showed no increased risk of death for naltrexone over placebo (RR 0.79, 95% CI 0.33–1.91). Although specified in the protocol, no meta-analysis of the specific SAEs due to cardiovascular or cerebrovascular events or cancers was undertaken owing to the low number of events recorded. Univariate and multivariate meta-regression analysis did not reveal any significance for any of the covariates.

#### Adverse events

A secondary analysis of 188 AEs (Additional file 5: Table S3) revealed only six statistically significant MedDRA preferred-term AEs. These were decreased appetite (RR 1.44, 95% CI 1.09–1.91), dizziness (RR 1.45, 95% CI 1.15–1.83), nausea (RR 1.59, 95% CI 1.37–1.84), sleepiness (RR 1.45, 95% CI 1.07–1.97), sweating (RR 1.89, 95% CI 1.25–2.87) and vomiting (RR 1.91, 95% CI 1.51–2.42). However, sensitivity analysis revealed these to be of only mild nature and common among all patients.

#### Withdrawals and withdrawals due to AEs

There was no evidence of a difference between naltrexone and placebo in the meta-analysis of RR of withdrawals (RR 0.99, 95% CI 0.93–1.05, *I*^2^ = 8%), whereas there was an increased risk of withdrawal due to AEs (RR 1.33, 95% CI 1.06–1.67, *I*^2^ = 0%).

#### Subgroup and sensitivity analyses

**Fig 3:**
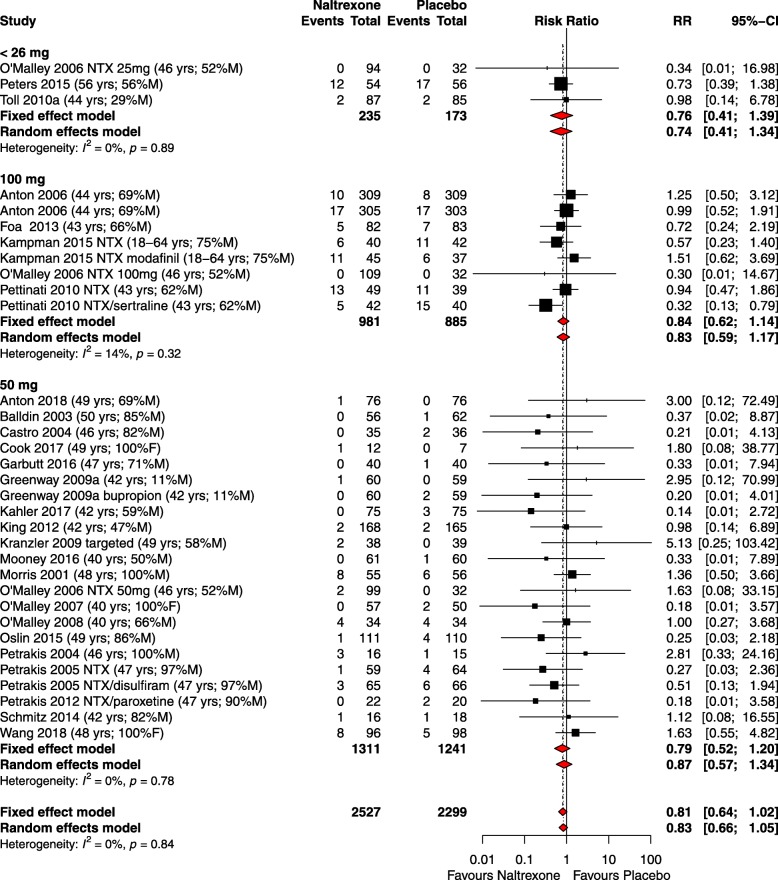
Forest plot of the subgroup analysis by dose of the risk ratio (RR) of serious adverse events in RCTs of naltrexone vs placebo. Data in parentheses show the mean or range of participants’ age and the percentage of male or female participants. Double zero studies (i.e. those which reported zero events in each treatment group) were excluded from the meta-analysis

In pre-specified subgroup analyses of RR of SAEs, there was no difference in results for different doses (Fig. 3) of naltrexone or for different disease groups/conditions. Because of the limited number of studies with dosages <26 mg compared to the other dosage groups, we specify that these results should be interpreted with caution. The assessment of SAEs by disease group is shown in Additional file 6: Figure S1. This analysis did not display any statistical significance. A post hoc analysis by length of study showed no difference in risk between studies of ≤15 weeks duration (RR 0.74, 95% CI 0.53–1.02, *I*^2^ = 0%) compared to studies >15 weeks duration (RR 0.96, 95% CI 0.69–1.34, *I*^2^ = 0%). Sensitivity analysis of the low risk of bias studies (RR 0.97, 95% CI 0.61–1.54, *I*^2^ = 0%) showed no difference in risk compared with studies at higher risk of bias (RR 0.80, 95% CI 0.61–1.05, *I*^2^ = 0%) (Additional file 7: Figure S2).

#### Assessment for publication bias

**Fig 4:**
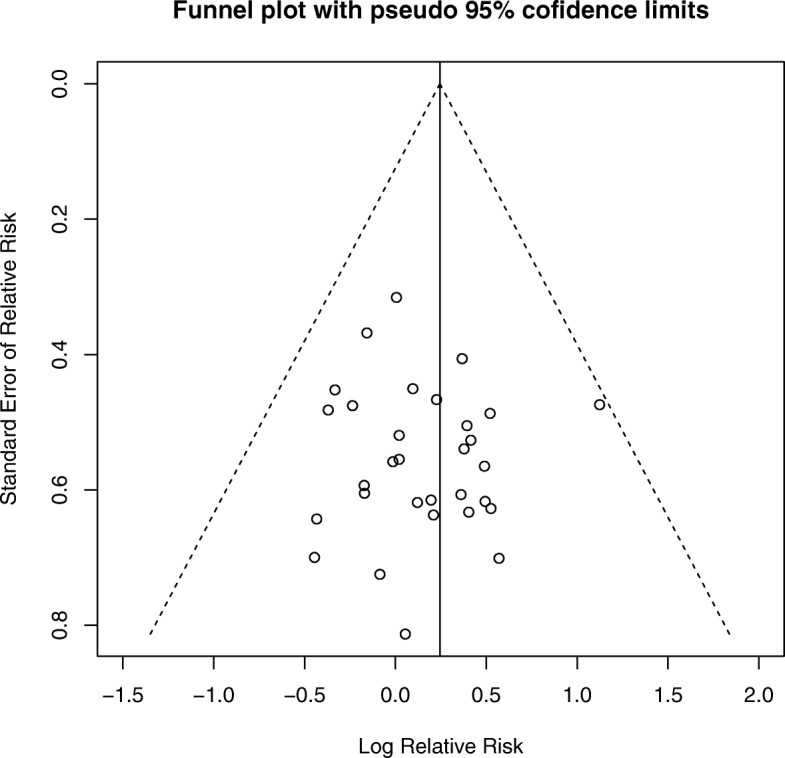
Funnel plot of weighted risk ratio (RR) of serious adverse events in RCTs of naltrexone vs placebo vs standard error

There was no evidence of funnel plot asymmetry to indicate publication bias for the RR of SAEs or for the RR of withdrawals or withdrawals due to AEs. The funnel plot for the main analysis is included as Fig. 4.

## Discussion

### Summary of main findings

This meta-analysis of 89 RCTs based on 11,194 participants showed no evidence of an increased risk of SAEs occurring for naltrexone compared to placebo. These findings were consistent across trials with varying duration, dosages and index conditions, suggesting that naltrexone is safe to use across a wide variety of licensed and non-licensed indications. We found that AEs such as dizziness, nausea and vomiting are potentially more common for naltrexone compared to placebo. However, this finding should be interpreted with caution because data reporting for AEs was poor (fewer than 21 studies contributed to the AE analyses).

### Strengths and limitations

There were several strengths of this review. One was the size, which was sufficiently large in both number of participants and number of studies that it would have enabled the detection of specific harms due to a drug. Papanikolaou and Ioannidis [59] calculated the sample size of a systematic review needed to detect a rare event (0.25%) occurring in about 1% of subjects as 4000 subjects (80% power and α = 0.05), and this systematic review contained over 10,000 subjects from 89 studies. In addition, this review included a broad range of studies from different countries, settings and disease groups, including patients with multiple morbidities or addictions. These latter complex scenarios more closely reflect clinical practice than the usual restrictive entry criteria of clinical trials. Hence, the relative effect size found is likely to be generalisable [60]. Our methodology for examining the outcome measures which were not the primary outcome measures in any of the clinical trials but are now part of the standard reporting of clinical trials reduced the risk of reporting and publication bias [61], as did the use of clinical trials registries.

It is likely that some studies inadequately reported and/or recorded SAEs. Therefore we checked and recorded any instances of discrepancies in data similar to previous reports [62]. We consider it unlikely that the missing or mis-recorded SAEs would have changed the conclusions of the meta-analysis, because there were no systematic differences between those studies adequately and inadequately reporting SAEs, and because the sensitivity analyses, particularly that including only studies with an overall low risk of bias, supported the main conclusion. There could have been under-recording of SAEs in studies with high attrition rates if follow-up was poor. Additionally, because adherence to the CONSORT extension for harms recommendations [33] was poor in many studies, particularly in the use of standardised definitions and the descriptions of events, we were unable to undertake any qualitative analysis of results.

This review was limited to studies of oral naltrexone, excluding studies involving current or prior opioid addiction or use. Our assessment of SAEs by disease group should only be considered as exploratory because classifying the populations into specific disease groups was not clear-cut owing to the predominance of AUDs even in studies of other disorders.

While the primary aim of this study was to examine SAE data from RCTs, we did examine AEs in a secondary analysis, but this analysis was based on limited data identified in the journal publication and the registry report. Previous evidence has also shown that the assessment and reporting of AEs is often inconsistent and incomplete across the studies. For example, a large safety review of 44 studies [63] of naltrexone for AUDs found that AEs were often not collected using standardised measures, that the methods for systematically capturing AEs were often not reported, and the reporting of AEs was highly selective.

Recording of AEs can be hampered by the presence of nocebo (harmful) effects (i.e. worsening symptoms during placebo treatment), which can vary disease by disease. Particularly in alcohol and drug addiction, placebo and nocebo mechanisms could impact on the therapeutic outcomes and side effects of treatments [64]. Although less likely in the recording of SAEs owing to their seriousness [64], this may have also impacted our results.

Finally, a few refinements to the protocol were necessary, but these occurred as recommended before any data collection occurred [65]. The main change was the exclusion of laboratory-based studies, studies of less than 4 weeks duration and cross-over studies from the review. The initial scoping exercise had not revealed the large numbers of such studies and attempting an analysis of all these would have exceeded available resources.

### Comparison with the existing literature

To our knowledge, this is the first large systematic review of SAEs in people taking naltrexone, excluding only those people taking opioids. Two large previous systematic reviews of naltrexone in AUDs were conducted by Rösner et al. [66] for the Cochrane Collaboration and Jonas et al. [63] for the Agency for Healthcare Research and Quality. Both examined AEs, but in fewer studies. Rösner et al. [66] analysed nine studies including two using injectable naltrexone, and calculated the RD of experiencing SAEs as −0.02 (95% CI −0.05–0.00). By using a wider range of studies and inclusion criteria and limiting the publication dates after the 1^st^ of January 2001, this review was able to provide a more accurate assessment of the risk of SAEs than any previous review.

### Implications for researchers, clinicians and policy makers

The results of this review are supportive of the wider use of naltrexone and have the realistic potential to impact on clinical guidelines. Policy makers (e.g. US Preventative Task Force and the National Institute of Clinical Excellence) are encouraged to use the findings of this review in conjunction with other studies focussed on benefits and cost-effectiveness of naltrexone to draw/revise evidence-based recommendations regarding the licensed use of naltrexone in a broader range of conditions. Treatment of AUDs, for which naltrexone is currently under-utilised, is a key area of consideration. Estimates suggest that about 58% of alcohol-dependent people in England want to reduce their drinking [67]. The increased use of pharmacotherapy for AUDs has been shown to be cost-effective and could reduce deaths [68–70].

This review shows the advantages of examining both benefit and risk profiles for drugs and the need for consistent and adequate recording of AEs and SAEs in reports of RCTs. Recent studies included in this review still did not consistently report harms to the standard suggested in the CONSORT extension for harms [33, 71], and differences in judgements on what constituted an SAE were evident between studies. Research on the efficacy of naltrexone for most diseases apart from AUDs and opioid abuse is currently lacking; naltrexone would seem an excellent candidate for repurposing given it is both safe and cheap, being long out of patent. It is also possible that naltrexone could be associated with changes in the rates of cancers and cardiovascular or cerebrovascular events given the complex interactions of opioids in the body [72, 73]. Thus, both large-scale pragmatic clinical trials of potentially new indications for naltrexone, and systematic evaluations through pharmaco-epidemiological studies using long-term safety data (e.g. the UK Clinical Practice Research Datalink (https://www.cprd.com/home/) are needed.

## Conclusions

This systematic review and meta-analysis found no evidence of a difference in risk of SAEs for oral naltrexone compared to placebo. This evidence supports the use of naltrexone in its currently licensed form and provides solid support to contemporary efforts studying naltrexone where it is currently unlicensed.

Box 1 Definitions of harms connected to the use of drugs in clinical trials The International Conference on Harmonisation of Technical Requirements for Registration of Pharmaceuticals for Human Use (ICH) [50] developed definitions for use in clinical trials which were then incorporated into EU [48] and US [49] law. The ICH (1994) gave the following definitions:

AE: Adverse event. An AE is defined as “Any untoward medical occurrence in a patient or clinical trial subject administered a medicinal product and which does not necessarily have a causal relationship with this treatment. An adverse event can therefore be any unfavourable and unintended sign (including an abnormal laboratory finding, for example), symptom or disease temporally associated with the use of a medicinal product, whether or not considered related to the medicinal product”.

SAE: Serious adverse event. An SAE is defined as “Any untoward medical occurrence or effect that at any dose results in death, is life-threatening, requires hospitalisation or prolongation of existing hospitalisation, results in persistent or significant disability or incapacity, or is a congenital anomaly or birth defect”.

These characteristics/consequences have to be considered at the time of the event. For example, regarding a life-threatening event, this refers to an event in which the subject was at risk of death at the time of the event; it does not refer to an event which hypothetically might have caused death if it were more severe.

Some medical events may jeopardise the subject or may require an intervention to prevent one of the above characteristics/consequences. Such events (hereinafter referred to as ‘important medical events’) should also be considered as ‘serious’ in accordance with the definition. (US regulations state “…*and* may require…” [49] rather than “…*or* may require…”).

The regulations state that “the judgement as to whether the event is serious is usually made by the reporting investigator” [48] and that “the assessment of whether there is a reasonable possibility of a causal relationship is usually made by the investigator” [48]. In this systematic review all judgements on seriousness and causality by study authors were therefore accepted.

The ICH differentiates seriousness, as defined above, from severity, which relates to the intensity of an event. AEs may be severe but relatively minor, for example, a severe headache. Regulations, and this systematic review, are only concerned with serious events.

ADR: Adverse drug reaction. All noxious and unintended responses to a medicinal product related to any dose.

Side effect: Negative (unfavourable) or positive (favourable) effects of a drug.

In randomised controlled trials, such definitions are best avoided as they require an assessment of causal link between an AE and the drug and hence could result in biased data collection [33].

## Additional files

Additional file 1: Specific search strategies for databases (DOCX 23 kb)

Additional file 2: Eligible studies for the review (DOCX 32 kb)

Additional file 3: **Table S1.** Characteristics of included studies (DOCX 43 kb)

Additional file 4: **Table S2.** Risk of bias summary (PDF 420 kb)

Additional file 5: **Table S3.** Meta-analysis of adverse events data across trials (DOCX 24 kb)

Additional file 6: **Figure S1.** Subgroup analysis of SAEs by disease type (PDF 13 kb)

Additional file 7: **Figure S2.** Forest plot of SAEs by risk of bias (PDF 13 kb)

## Abbreviations

AE: Adverse event
AUD: Alcohol use disorder
CENTRAL: Cochrane Central Register of Controlled Trials
CI: Confidence interval
CONSORT: Consolidated Standards of Reporting Trials
CT-3: “Detailed guidance on the collection, verification and presentation of adverse event/ reaction reports arising from clinical trials on medicinal products for human use (CT-3)”, published by the European Commission 2011
EudraCT: European Clinical Trials Database
FDA: US Food and Drug Administration
HIV: Human immunodeficiency virus
ICH: International Conference on Harmonisation of Technical Requirements for Registration of Pharmaceuticals for Human Use
LDN: Low dose naltrexone
PRISMA: Preferred Reporting Items for Systematic Reviews and Meta-analyses
PROSPERO: International Prospective Register of Systematic Reviews
RCT: Randomised controlled trial
RD: Risk difference
RR: Risk ratio or relative risk
SAE: Serious adverse event
