# Supplementary material for: Serious adverse events reported in placebo randomised controlled trials of oral naltrexone: a systematic review and meta-analysis
Source: BMC Med. 2019 Jan 15;17:10. doi: 10.1186/s12916-018-1242-0 (PMC6332608; doi:10.1186/s12916-018-1242-0)
Supplement: Supplementary file 1 — Specific search strategies for databases (DOCX 23 kb) [file 12916_2018_1242_MOESM1_ESM.docx]

**Additional file 1: Specific search strategies for databases**

**CENTRAL**

“naltrexone” in Title, Abstract, Keywords, Publication year from 2001 to 2018 in Trials (word variations have been searched).

Items found = 1131

**Pubmed Medline**

The Cochrane highly sensitive search strategy for randomised trials was used, combined with naltrexone [MeSH descriptor explode all trees] OR naltrexone [tiab] for the years 2001 to 2018.

| Search | Query |
| --- | --- |
| #1 | randomized controlled trial[Publication Type] |
| #2 | controlled clinical trial[Publication Type] |
| #3 | randomized[Title/Abstract] |
| #4 | placebo[Title/Abstract] |
| #5 | drug therapy [subheading] |
| #6 | randomly[Title/Abstract] |
| #7 | trial[Title/Abstract] |
| #8 | groups[Title/Abstract] |
| #9 | (#1 OR #2 OR #3 OR #4 OR #5 OR #6 OR #7 or #8) |
| #10 | (animals [MeSH terms] NOT humans [MeSH terms]) |
| #11 | (#9 NOT #10) |
| #12 | "naltrexone" [all fields] |
| #13 | (#11 AND #12) |
| #14 | #13 limit to 2001/01/01 to 2018/05/01 |

**Ovid Embase**

The Cochrane highly sensitive search strategy was used, which is available as a search strategy on the Ovid platform. This was combined with naltrexone as a subject heading (exploded) and as a keyword and limited to the years 2001 to current.

| Search | query |  |
| --- | --- | --- |
| 1 | Clinical Trial.pt |  |
| 2 | randomized.tw |  |
| 3 | placebo.tw |  |
| 4 | dt.fs |  |
| 5 | randomly.tw |  |
| 6 | trial.tw |  |
| 7 | groups.tw |  |
| 8 | or/1-7 |  |
| 9 | (animals not humans).sh |  |
| 10 | 8 not 9 |  |
| 11 | naltrexone.mp [mp=title, subject heading word, registry word, abstract, trade name/generic name] or exp. naltrexone/ |  |
| 12 | limit 11 to yr="2001-Current" |  |
| 13 | 10 and 12 |  |
|  |  |  |

PsychINFO

The Cochrane highly sensitive search strategy which is available as a search strategy on the Ovid platform was combined with naltrexone as a subject heading (exploded) and as a keyword and limited to the years 2001 to current.

| Search | query |
| --- | --- |
| 1 | Clinical Trial.pt |
| 2 | randomized.tw |
| 3 | placebo.tw |
| 4 | dt.fs |
| 5 | randomly.tw |
| 6 | trial.tw |
| 7 | groups.tw |
| 8 | or/1-7 |
| 9 | (animals not humans).sh |
| 10 | 8 not 9 |
| 11 | naltrexone.mp [mp=title, subject heading word, registry word, abstract, trade name/generic name] or exp. naltrexone/ |
| 12 | limit 11 to yr="2001-Current" |
| 13 | 10 and 12 |

**International Pharmaceutical Abstracts**

The Cochrane highly sensitive search strategy which is available as a search strategy on the Ovid platform was combined with naltrexone as a subject heading, limited to the years 2001 to current

| Search | query |  |
| --- | --- | --- |
| 1 | Clinical Trial.pt |  |
| 2 | randomized.tw |  |
| 3 | placebo.tw |  |
| 4 | dt.fs |  |
| 5 | randomly.tw |  |
| 6 | trial.tw |  |
| 7 | groups.tw |  |
| 8 | or/1-7 |  |
| 9 | (animals not humans).sh |  |
| 10 | 8 not 9 |  |
| 11 | naltrexone.mp [mp=title, subject heading word, registry word, abstract, trade name/generic name] |  |
| 12 | limit 11 to yr="2001-Current" |  |
| 13 | 10 and 12 |  |
|  |  |  |
