# Supplementary material for: Serious adverse events reported in placebo randomised controlled trials of oral naltrexone: a systematic review and meta-analysis
Source: BMC Med. 2019 Jan 15;17:10. doi: 10.1186/s12916-018-1242-0 (PMC6332608; doi:10.1186/s12916-018-1242-0)
Supplement: Supplementary file 2 — Eligible studies for the review (DOCX 32 kb) [file 12916_2018_1242_MOESM2_ESM.docx]

**Additional file 2: Eligible studies for the review**

Abou-Raya 2013

Abou-Raya S, Abou-Raya A, Khadrawi T. Efficacy of naltrexone in the treatment of fibromyalgia: randomized controlled trial. Ann Rheum Dis. 2013;72(Suppl. 3):719.

Ahmadi 2004

Ahmadi J, Babaeebeigi M, Maany I, Porter J, Mohagheghzadeh M, Ahmadi N, et al. Naltrexone for alcohol-dependent patients. Ir J Med Sci. 2004;173(1):34–7.

Anton 2005

Anton RF, Moak DH, Latham P, Waid LR, Myrick H, Voronin K, et al. Naltrexone combined with either cognitive behavioral or motivational enhancement therapy for alcohol dependence. J Clin Psychopharmacol. 2005;25(4):349–57.

Anton 2006

Anton RF, O’Malley SS, Ciraulo DA, Cisler RA, Couper D, Donovan DM, et al. Combined pharmacotherapies and behavioral interventions for alcohol dependence: the COMBINE study: a randomized controlled trial. JAMA. 2006;295(17):2003–17.

Anton 2011a

Anton RF, Myrick H, Wright TM, Latham PK, Baros AM, Waid LR, et al. Gabapentin combined with naltrexone for the treatment of alcohol dependence. Am J Psychiatry. 2011;168(7):709–17.

Anton 2011b

Anton RF, Baros A, Latham P, Randall P, Stewart S, Vergne D. Naltrexone plus aripiprazole compared to naltrexone alone and placebo in the treatment of alcohol dependence - a double blind pilot study. Neuropsychopharmacology. 2011;36:S234–5.

Anton 2018

Anton RF, Latham PK, Voronin KE, Randall PK, Book SW, Hoffman M, et al. Nicotine-use/smoking is associated with the efficacy of naltrexone in the treatment of alcohol dependence. Alcohol Clin Exp Res. 2018;42(4):751–60.

Balldin 2003

Balldin J, Berglund M, Borg S, Månsson M, Bendtsen P, Franck J, et al. A 6-month controlled naltrexone study: combined effect with cognitive behavioral therapy in outpatient treatment of alcohol dependence. Alcohol Clin Exp Res. 2003;27(7):1142–9.

Baltieri 2008

Baltieri DA, Daró FR, Ribeiro PL, De Andrade AG. Comparing topiramate with naltrexone in the treatment of alcohol dependence. Addiction. 2008;103(12):2035–44.

Batki 2009

Batki SL, Dimmock JA, Ploutz-Snyder R, Meszaros ZS, Canfield K. Directly monitored naltrexone reduces heavy drinking in schizophrenia: preliminary analysis of a controlled trial. Alcohol Clin Exp Res. 2009;33(6, Sp. Iss. S1):113A.

Brown 2009

Brown ES, Carmody TJ, Schmitz JM, Caetano R, Adinoff B, Swann AC, et al. A randomized, double-blind, placebo-controlled pilot study of naltrexone in outpatients with bipolar disorder and alcohol dependence. Alcohol Clin Exp Res. 2009;33(11):1863–9.

Byars 2005

Byars JA, Frost-Pineda K, Jacobs WS, Gold MS. Naltrexone augments the effects of nicotine replacement therapy in female smokers. J Addict Dis. 2005;24(2):49–60.

Castro 2004

Castro LAP de G. Ensaio clínico duplo-cego randomizado e placebo-controlado com naltrexona associado à intervenção breve no tratamento ambulatorial da dependência de álcool/. Unpublished Dissertation:PhD Federal University of São Paulo. Universidade Federal de São Paulo.; 2004.

COMBINE Study Research Group 2003

COMBINE Study Research Group. Testing combined pharmacotherapies and behavioral interventions for alcohol dependence (the COMBINE Study): a pilot feasibility study. Alcohol Clin Exp Res. 2003;27(7):1123–31.

Cook 2017

Cook RL, Weber KM, Mai D, Thoma K, Hu X, Brumback B, et al. Acceptability and feasibility of a randomized clinical trial of oral naltrexone vs. placebo for women living with HIV infection: study design challenges and pilot study results. Contemp Clin Trials. 2017;60:72–7.

Davidson 2004

Davidson D, Saha C, Scifres S, Fyffe J, O’Connor S, Selzer C. Naltrexone and brief counseling to reduce heavy drinking in hazardous drinkers. Addict Behav. 2004;29(6):1253–8.

Foa 2013

Foa EB, Yusko DA, McLean CP, Suvak MK, Bux DA, Oslin D, et al. Concurrent naltrexone and prolonged exposure therapy for patients with comorbid alcohol dependence and PTSD. JAMA. 2013;310(5):488–95.

Fogaça 2011

Fogaça MN, Santos-Galduróz RF, Eserian JK, Galduróz JCF. The effects of polyunsaturated fatty acids in alcohol dependence treatment--a double-blind, placebo-controlled pilot study. BMC Clin Pharmacol. 2011;11:10.

Garbutt 2010

Garbutt J, Kalka-Juhl L, Kampov-Polevoy A, Wells S, Nicholas L, Gallop R, et al. Feasibility and tolerability of a combination of naltrexone and baclofen for alcohol dependence: a pilot study. Alcohol Clin Exp Res. 2010;34(s2):178a.

Garbutt 2016

Garbutt JC, Kampov-Polevoy AB, Kalka-Juhl LS, Gallop RJ. Association of the sweet-liking phenotype and craving for alcohol with the response to naltrexone treatment in alcohol dependence. JAMA Psychiatry. 2016;73(10):1056–63.

Gastpar 2002

Gastpar M, Bonnet U, Böning J, Mann K, Schmidt LG, Soyka M, et al. Lack of efficacy of naltrexone in the prevention of alcohol relapse: results from a German multicenter study. J Clin Psychopharmacol. 2002;22(6):592–8.

Grant 2008

Grant JE, Kim SW, Hartman BK. A double-blind, placebo-controlled study of the opiate antagonist naltrexone in the treatment of pathological gambling urges. J Clin Psychiatry. 2008;69(5):783–9.

Grant 2009

Grant JE, Kim SW, Odlaug BL. A double-blind, placebo-controlled study of the opiate antagonist, naltrexone, in the treatment of kleptomania. Biol Psychiatry. 2009;65(7):600–6.

Grant 2012

Grant JE. Double-blind naltrexone in compulsive sexual behavior NCT00467558 [Internet]. ClinicalTrials.gov NCT00467558. 2012 [cited 2017 Jul 7]. Available from: <https://clinicaltrials.gov/ct2/show/study/NCT00467558?term=NCT00467558&rank=1>

Grant 2014

Grant JE, Odlaug BL, Schreiber LRN, Kim SW. The opiate antagonist, naltrexone, in the treatment of trichotillomania. Results of a double-blind, placebo-controlled study. J Clin Psychopharmacol. 2014;34(1):134–8.

Greenway 2009a

Greenway FL, Whitehouse MJ, Guttadauria M, Anderson JW, Atkinson RL, Fujioka K, et al. Rational design of a combination medication for the treatment of obesity. Obesity. 2009;17(1):30–9.

Greenway 2009b

Greenway FL, Dunayevich E, Tollefson G, Erickson J, Guttadauria M, Fujioka K, et al. Comparison of combined bupropion and naltrexone therapy for obesity with monotherapy and placebo. J Clin Endocrinol Metab. 2009;94(12):4898–906.

Guardia 2002

Guardia J, Caso C, Arias F, Gual A, Sanahuja J, Ramírez M, et al. A double-blind, placebo-controlled study of naltrexone in the treatment of alcohol-dependence disorder: results from a multicenter clinical trial. Alcohol Clin Exp Res. 2002;26(9):1381–7.

Heinälä 2001

Heinälä P, Alho H, Kiianmaa K, Lönnqvist J, Kuoppasalmi K, Sinclair JD. Targeted use of naltrexone without prior detoxification in the treatment of alcohol dependence: a factorial double-blind, placebo-controlled trial. J Clin Psychopharmacol. 2001;21(3):287–92.

Huang 2002

Huang X, Huang X, Peng H, Mai G. [Placebo-controlled trial of naltrexone in outpatient treatment of alcohol dependence] (Chinese). Chinese Ment Heal J. 2002;16(5):302–295.

Huang 2005

Huang M-C, Chen C-H, Yu J-M, Chen C-C. A double-blind, placebo-controlled study of naltrexone in the treatment of alcohol dependence in Taiwan. Addict Biol. 2005;10(3):289–92.

Jayaram-Lindström 2008

Jayaram-Lindström N, Hammarberg A, Beck O, Franck J. Naltrexone for the treatment of amphetamine dependence: a randomized, placebo-controlled trial. Am J Psychiatry. 2008;165(11):1442–8.

Kahler 2017

Kahler CW, Cioe PA, Tzilos GK, Spillane NS, Leggio L, Ramsey SE, et al. A double-blind randomized placebo-controlled trial of oral naltrexone for heavy-drinking smokers seeking smoking cessation treatment. Alcohol Clin Exp Res. 2017;41(6):1201–11.

Kampman 2015

Kampman K, Pettinati H, Lynch K, Plebani J, Lachewitz J, Feeney K, et al. Modafinil and naltrexone for the treatment of comorbid cocaine and alcohol dependence. Drug Alcohol Depend. 2015;146:e152.

Kiefer 2003

Kiefer F, Jahn H, Tarnaske T, Helwig H, Briken P, Holzbach R, et al. Comparing and combining naltrexone and acamprosate in relapse prevention of alcoholism: a double-blind placebo-controlled study. Arch Gen Psychiatry. 2003;60(1):92–9.

Killeen 2004

Killeen TK, Brady KT, Gold PB, Simpson KN, Faldowski RA, Tyson C, et al. Effectiveness of naltrexone in a community treatment program. Alcohol Clin Exp Res. 2004;28(11):1710–7.

Kim 2001

Kim SW, Grant JE, Adson DE, Shin YC. Double-blind naltrexone and placebo comparison study in the treatment of pathological gambling. Biol Psychiatry. 2001;49(11):914–21.

King 2006

King A, de Wit H, Riley R, Cao D, Niaura R, Hatsukami D. Efficacy of naltrexone in smoking cessation: a preliminary study and an examination of sex differences. Nicotine Tob Res. 2006;8(5):671–82.

King 2012

King AC, Cao D, O’Malley SS, Kranzler HR, Cai X, DeWit H, et al. Effects of naltrexone on smoking cessation outcomes and weight gain in nicotine-dependent men and women. J Clin Psychopharmacol. 2012;32(5):630–6.

Kovanen 2016

Kovanen L, Basnet S, Castr??n S, Pankakoski M, Saarikoski ST, Partonen T, et al. A randomised, double-blind, placebo-controlled trial of as-needed naltrexone in the treatment of pathological gambling. Eur Addict Res. 2016;22(2):70–9.

Kranzler 2003

Kranzler HR, Armeli S, Tennen H, Blomqvist O, Oncken C, Petry N, et al. Targeted naltrexone for early problem drinkers. J Clin Psychopharmacol. 2003;23(3):294–304.

Kranzler 2009

Kranzler HR, Armeli S, Tennen H, Chan G, Covault J, Arias A, et al. Targeted naltrexone for problem drinkers. J Clin Psychopharmacol. 2009;29(4):350–7.

Krishnan-Sarin 2003

Krishnan-Sarin S, Meandzija B, O’Malley S. Naltrexone and nicotine patch in smoking cessation: a preliminary study. Nicotine Tob Res. 2003;5(6):851–7.

Krystal 2001

Krystal JH, Cramer JA, Krol WF, Kirk GF, Rosenheck RA. Naltrexone in the treatment of alcohol dependence. N Engl J Med. 2001;345(24):1734–9.

Latt 2002

Latt NC, Jurd S, Houseman J, Wutzke SE. Naltrexone in alcohol dependence: a randomised controlled trial of effectiveness in a standard clinical setting. Med J Aust. 2002;176(11):530–4.

Lee 2001

Lee A, Tan S, Lim D, Winslow RM, Wong KE, Allen J, et al. Naltrexone in the treatment of male alcoholics — an effectiveness study in Singapore. Drug Alcohol Rev. 2001;20(2):193–9.

Mann 2013

Mann K, Lemenager T, Hoffmann S, Reinhard I, Hermann D, Batra A, et al. Results of a double-blind, placebo-controlled pharmacotherapy trial in alcoholism conducted in Germany and comparison with the US COMBINE study. Addict Biol. 2013;18(6):937–46.

Monterosso 2001

Monterosso JR, Flannery BA, Pettinati HM, Oslin DW, Rukstalis M, O’Brien CP, et al. Predicting treatment response to naltrexone: the influence of craving and family history. Am J Addict. 2001;10(3):258–68.

Mooney 2016

Mooney ME, Schmitz JM, Allen S, Grabowski J, Pentel P, Oliver A, et al. Bupropion and naltrexone for smoking cessation: a double-blind randomized placebo-controlled clinical trial. Clin Pharmacol Ther. 2016;100(4):344–52.

Morgenstern 2012

Morgenstern J, Kuerbis AN, Chen AC, Kahler CW, Bux DA, Kranzler HR. A randomized clinical trial of naltrexone and behavioral therapy for problem drinking men who have sex with men. J Consult Clin Psychol. 2012;80(5):863–75.

Morley 2006

Morley KC, Teesson M, Reid SC, Sannibale C, Thomson C, Phung N, et al. Naltrexone versus acamprosate in the treatment of alcohol dependence: a multi-centre, randomized, double-blind, placebo-controlled trial. Addiction. 2006;101(10):1451–62.

Morris 2001

Morris PL, Hopwood M, Whelan G, Gardiner J, Drummond E. Naltrexone for alcohol dependence: a randomized controlled trial. Addiction. 2001;96(11):1565–73.

Murphy 2014

Murphy BL, Ravichandran C, Babb SM, Cohen BM. Naltrexone in bipolar disorder with depression: a double-blind, placebo-controlled study. J Clin Psychopharmacol. 2014;34(6):749–51.

Niederhofer 2003

Niederhofer H, Staffen W, Mair A. Comparison of naltrexone and placebo in treatment of alcohol dependence of adolescents. Alcohol Treat Q. 2003;21(2):87–95.

O’Malley 2006

O’Malley SS, Cooney JL, Krishnan-Sarin S, Dubin JA, McKee SA, Cooney NL, et al. A controlled trial of naltrexone augmentation of nicotine replacement therapy for smoking cessation. Arch Intern Med. 2006;166(6):667–74.

O’Malley 2007

O’Malley SS, Sinha R, Grilo CM, Capone C, Farren CK, McKee SA, et al. Naltrexone and cognitive behavioral coping skills therapy for the treatment of alcohol drinking and eating disorder features in alcohol-dependent women: a randomized controlled trial. Alcohol Clin Exp Res. 2007;31(4):625–34.

O’Malley 2008

O’Malley SS, Robin RW, Levenson AL, Greywolf I, Chance LE, Hodgkinson CA, et al. Naltrexone alone and with sertraline for the treatment of alcohol dependence in Alaska natives and non-natives residing in rural settings: a randomized controlled trial. Alcohol Clin Exp Res. 2008;32(7):1271–83.

O’Malley 2015

O’Malley SS, Corbin WR, Leeman RF, DeMartini KS, Fucito LM, Ikomi J, et al. Reduction of alcohol drinking in young adults by naltrexone: a double-blind, placebo-controlled, randomized clinical trial of efficacy and safety. J Clin Psychiatry. 2015;76(10):207–13.

Orexigen OT-101 2010

Orexigen Therapeutics Inc. Contrave (naltrexone SRbupropion SR combination). Advisory Committee briefing document NDA 200063. US Food and Drug Administration Endocrinological and Metabolics Drugs Advisory Committee Meeting December 7 2010 pp 40, 46, 48, 49, 95, 239

Oslin 2005

Oslin DW. Treatment of late-life depression complicated by alcohol dependence. Am J Geriatr Psychiatry. 2005;13(6):491–500.

Oslin 2008

Oslin DW, G. LK, Pettinati HM, Kampmann KM, Gariti P, Gelfand L, et al. A placebo-controlled randomized clinical trial of naltrexone in the context of different levels of psychosocial intervention. Alcohol Clin Exp Res. 2008;32(7):1299–308.

Oslin 2015

Oslin DW, Leong SH, Lynch KG, Berrettini W, O’Brien CP, Gordon AJ, et al. Naltrexone vs placebo for the treatment of alcohol dependence. A randomized clinical trial. JAMA Psychiatry. 2015;72(5):430–7.

Papay 2014

Papay K, Xie SX, Stern M, Hurtig H, Siderowf A, Duda JE, et al. Naltrexone for impulse control disorders in Parkinson disease: a placebo-controlled study. Neurology. 2014;83(9):826–33.

Peters 2015

Peters K, Affronti M, Woodring S, Lipp Er, Healy P, McSherry F, et al. Low-dose naltrexone in newly diagnosed high-grade glioma: placebo-controlled, double-blind, randomized pilot study. Neuro Oncol. 2015;17(suppl 5):v192.

Petrakis 2004

Petrakis IL, O’Malley S, Rounsaville B, Poling J, McHugh-Strong C, Krystal JH. Naltrexone augmentation of neuroleptic treatment in alcohol abusing patients with schizophrenia. Psychopharmacology (Berl). 2004;172(3):291–7.

Petrakis 2005

Petrakis IL, Poling J, Levinson C, Nich C, Carroll K, Rounsaville B. Naltrexone and disulfiram in patients with alcohol dependence and comorbid psychiatric disorders. Biol Psychiatry. 2005;57(10):1128–37.

Petrakis 2012

Petrakis IL, Ralevski E, Desai N, Trevisan L, Gueorguieva R, Rounsaville B, et al. Noradrenergic vs serotonergic antidepressant with or without naltrexone for veterans with PTSD and comorbid alcohol dependence. Neuropsychopharmacology. 2012;37(4):996–1004.

Pettinati 2008a

Pettinati HM, Kampman KM, Lynch KG, Xie H, Dackis C, Rabinowitz AR, et al. A double blind, placebo-controlled trial that combines disulfiram and naltrexone for treating co-occurring cocaine and alcohol dependence. Addict Behav. 2008;33(5):651–67.

Pettinati 2008b

Pettinati HM, Kampman KM, Lynch KG, Suh JJ, Dackis CA, Oslin DW, et al. Gender differences with high-dose naltrexone in patients with co-occurring cocaine and alcohol dependence. J Subst Abuse Treat. 2008;34(4):378–90.

Pettinati 2010

Pettinati HM, Oslin David W, Kampmann KM, Dundon WD, Xie H, Gallis TL, et al. A double blind, placebo-controlled trial that combines sertraline and naltrexone for treating co-occurring depression and alcohol dependence. Am J Psychiatry. 2010;167(6):668–75.

Potenza 2017

Potenza M. Investigation of naltrexone for pathological gambling [Internet]. Clinicaltrials.gov NCT01057862. 2017 [cited 2018 May 25]. Available from: <https://clinicaltrials.gov/ct2/show/study/NCT01057862?term=NCT01057862&rank=1>

Salloum 2011

Salloum IM, Cornelius JR, Douaihy A, Caceda R, Miao F, Levent K, et al. Longstanding cannabis abuse is associated with decreased likelihood of remission from major depression. Neuropsychopharmacology. 2011;36:S300–1.

Salloum 2014

Salloum IM, Bellon A, Hyman S, Miao F, Andreev V. Naltrexone added to valproate decreases alcohol use in patients with alcoholism and biploar disorders: preliminary findings. Alcohol Clin Exp Res. 2014;38:138a.

Santos 2016

Santos G-M, Coffin P, Santos D, Huffaker S, Matheson T, Euren J, et al. Feasibility, acceptability, and tolerability of targeted naltrexone for nondependent methamphetamine-using and binge-drinking men who have sex with men. JAIDS J Acquir Immune Defic Syndr. 2016;72(1):21–30.

Schmitz 2001

Schmitz JM, Stotts AL, Rhoades HM, Grabowski J. Naltrexone and relapse prevention treatment for cocaine dependent patients. Addict Behav. 2001;26(2):167–80.

Schmitz 2004

Schmitz JM, Stotts AL, Sayre SL, DeLaune KA, Grabowski J. Treatment of cocaine–alcohol dependence with naltrexone and relapse prevention therapy. Am J Addict. 2004;13(4):333–41.

Schmitz 2009

Schmitz JM, Lindsay JA, Green CE, Herin D V., Stotts AL, Moeller FG. High-dose naltrexone therapy for cocaine-alcohol dependence. Am J Addict. 2009;18(5):356–62.

Schmitz 2014

Schmitz JM, Green CE, Stotts AL, Lindsay JA, Rathnayaka NS, Grabowski J, et al. A two-phased screening paradigm for evaluating candidate medications for cocaine cessation or relapse prevention: modafinil, levodopa-carbidopa, naltrexone. Drug Alcohol Depend. 2014;136:100–7.

Seifrabiei 2008

Seifrabiei MA, Abbasi M, Montazeri A, Shahnazari F, Pooya A. Quality of life in hematologic cancer patients: a randomized clinical trial of low dose naltrexone versus placebo. Am J Appl Sci. 2008;5(7):872–5.

Smith 2011

Smith JP, Bingaman SI, Ruggiero F, Mauger DT, Mukherjee A, McGovern CO, et al. Therapy with the opioid antagonist natrexone promotes mucosal healing in active Crohn’s disease: a randomized placebo-controlled trial. Dig Dis Sci. 2011;56(7):2088–97.

Smith 2013

Smith JP, Field D, Bingaman SI, Evans R, Mauger D. Safety and tolerability of low dose naltrexone therapy in children with moderate to severe Crohn’s disease: a pilot study. J Clin Gastroenterol. 2013;47(4):339–45.

Spencer 2016

Spencer TJ. A double-blind comparison of naltrexone and placebo in adults with attention deficit hyperactivity disorder. [Internet]. ClinicalTrials.gov NCT01721330. 2016 [cited 2017 Jul 8]. Available from: <https://clinicaltrials.gov/ct2/show/study/NCT01721330?term=NCT01721330&rank=1>

Spencer 2018

Spencer TJ, Bhide P, Zhu J, Faraone S V., Fitzgerald M, Yule AM, et al. Opiate antagonists do not interfere with the clinical benefits of stimulants in ADHD: a double-blind placebo-controlled trial of the mixed opioid receptor antagonist naltrexone. J Clin Psychiatry. 2018;79(1):19–25.

Taveira 2014

Taveira TH, Wu W-C, Tschibelu E, Borsook D, Simonson DC, Yamamoto R, et al. The effect of naltrexone on body fat mass in olanzapine-treated schizophrenic or schizoaffective patients: a randomized double-blind placebo-controlled pilot study. J Psychopharmacol. 2014;28(4):395–400.

Tek 2014

Tek C, Ratliff J, Reutenauer E, Ganguli R, O’Malley SS. A randomized, double-blind, placebo-controlled pilot study of naltrexone to counteract antipsychotic-associated weight gain: proof of concept. J Clin Psychopharmacol. 2014 Oct;34(5):608–12.

Toll 2010a

Toll BA, White M, Wu R, Meandzija B, Jatlow P, Makuch R, et al. Low-dose naltrexone augmentation of nicotine replacement for smoking cessation with reduced weight gain: a randomized trial. Drug Alcohol Depend. 2010;111(3):200–6.

Toll 2010b

Toll BA, Wu R, Meandzija B, O’Malley SS. Naltrexone and varenicline: weight gain and tolerability in smokers. In: Society for Research on Nicotine and Tobacco 16th Annual Meeting 24th to 27th Feb 2010. Baltimore, Maryland, USA: Society for Research on Nicotine and Tobacco; 2010. p. 69, NaN-18.

Toneatto 2009

Toneatto T, Brands B, Selby P. A randomized, double-blind, placebo-controlled trial of naltrexone in the treatment of concurrent alcohol use disorder and pathological gambling. Am J Addict. 2009;18(3):219–25.

Wang 2018

Wang Y, Chen X, Hahn JA, Brumback B, Zhou Z, Miguez MJ, et al. Phosphatidylethanol in comparison to self-reported alcohol consumption among HIV-infected women in a randomized controlled trial of naltrexone for reducing hazardous drinking. Alcohol Clin Exp Res. 2018;42(1):128–34.
