# Supplementary material for: Serious adverse events reported in placebo randomised controlled trials of oral naltrexone: a systematic review and meta-analysis
Source: BMC Med. 2019 Jan 15;17:10. doi: 10.1186/s12916-018-1242-0 (PMC6332608; doi:10.1186/s12916-018-1242-0)
Supplement: Supplementary file 3 — Table S1. Characteristics of included studies (DOCX 43 kb) [file 12916_2018_1242_MOESM3_ESM.docx]

**Additional file 3: Table S1: Characteristics of Included Studies**

| **Study** | **Conditions studied** | **Country** | **Final dose of naltrexone** | **Length of study** | **Other drugs & therapies trialled** | **Age mean/ median** |
| --- | --- | --- | --- | --- | --- | --- |
| Abou-Raya 2013 | fibromyalgia | Egypt | 4.5 mg | 24 weeks |  | not stated |
| Ahmadi 2004 | alcohol | Iran | 50 mg | 36 weeks | counselling | 43 |
| Anton 2005 | alcohol | USA | 50 mg | 12 weeks | CBT or motivational enhancement therapy | 44 |
| Anton 2006 | alcohol | USA | 100 mg | 16 weeks | acamprosate, (4 arms, factorial) MM +/- CBI. (One arm CBI only, not included in analysis.) | median 44 |
| Anton 2011a | alcohol | USA | 50 mg | 16 weeks | 3 arms (gabapentin+NTX arm not included in this analysis), MM & CBI | 45 |
| Anton 2011b | alcohol | USA | 50 mg | 16 weeks | 3 arms (aripiprazole arm not included in analysis), MM | 48 |
| Anton 2018 | alcohol | USA | 50 mg | 16 weeks | MM | 49 |
| Balldin 2003 | alcohol | Sweden | 50 mg | 6 months | CBT or supportive therapy | 50 |
| Baltieri 2008 | alcohol | Brazil | 50 mg | 12 weeks | 3 arms (topiramate arm not included in analysis), relapse prevention counselling | 44 |
| Batki 2009 | alcohol and schizophrenia | USA | 50 mg averaged dose (directly observed intake 3 times weekly) | 12 weeks | motivational counselling | 42 |
| Brown 2009 | bipolar disorder & alcohol | USA | 50 mg | 12 weeks | CBT | 41 |
| Byars 2005 | smoking | USA | 50 mg | 12 weeks | NRT & psychosocial therapy | not stated – 18 to 65 |
| Castro 2004 | alcohol | Brazil | 50 mg | 12 weeks | brief intervention | 46 |
| Combine study research group 2003 | alcohol | USA | 100 mg | 16 weeks | acamprosate, (4 arm factorial), MM +/- CBI | 42 |
| Cook 2017 | alcohol and HIV | USA | 50 mg | 4 months | anti-retroviral treatment where appropriate clinically | 49 |
| Davidson 2004 | alcohol | USA | 50 mg | 10 weeks | brief counselling | 49 |
| Foa 2013 | PTSD & alcohol | USA | 100 mg | 24 weeks | supportive counselling +/- prolonged exposure therapy | 43 |
| Fogaca 2011 | alcohol | Brazil | 50 mg | 90 days | polyunsaturated fatty acids (PUFAs), (4 arms factorial) | 43 |
| Garbutt 2010 | alcohol | USA | 50 mg | 11 weeks | baclofen, (4 arms factorial), counselling | not stated - 25 to 60 |
| Garbutt 2016 | alcohol | \| USA \| \| --- \| | 50 mg | 12 weeks | counselling | 47 |
| Gastpar 2002 | alcohol | Germany | 50 mg | 12 weeks | psychosocial alcohol treatment program | 43 |
| Grant 2008 | pathological gambling | USA | 3 arms, 50 mg, 100 mg and 150 mg, results all combined so not possible to analyse separately | 18 weeks - 17 weeks double blind |  | 46 |
| Grant 2009 | kleptomania | USA | 150 mg | 8 weeks | (76% also on psychotropic medication) | 34 |
| Grant 2012 | compulsive sexual behaviour | USA | 150 mg | 8 weeks |  | 32 |
| Grant 2014 | trichotillomania | USA | 150 mg | 8 weeks |  | 33 |
| Greenway 2009a | obesity | USA | 50 mg | 16 or 24 weeks, depending on study arm | S.R. bupropion, (multi-arm factorial), minimal dietary advice | 42 |
| Greenway 2009b | obesity | USA | 16, 32 or 48 mg depending on study arm | 24 or 48 weeks, depending on study arm | S.R. bupropion | 45 |
| Guardia 2002 | alcohol | Spain | 50 mg | 12 weeks | supportive group therapy & individual counselling by physician | 42 |
| Heinala 2001 | alcohol | Finland | 50 mg | 32 weeks = 12 weeks plus 20 weeks targeted medication | group cognitive coping skills or group supportive therapy | 46 |
| Huang 2002 | alcohol | China | 30 mg | 12 weeks |  | 45 |
| Huang 2005 | alcohol | Taiwan | 50 mg | 14 weeks | supportive psychotherapy | 41 |
| Jayaram-Lindstrom 2008 | amphetamine | Sweden | 50 mg | 12 weeks | relapse prevention therapy | 40 |
| Kahler 2017 | smoking & alcohol | USA | 50 mg | 10 weeks | NRT & counselling for heavy drinking and smoking | 42 |
| Kampman 2015 | cocaine and alcohol | USA | 150 mg for males, 100 mg for females | 13 weeks | modafinil, (4 arm factorial) & CBT | not stated - 18 to 64 |
| Kiefer 2003 | alcohol | Germany | 50 mg | 12 weeks | acamprosate, (4 arm factorial), abstinence orientated group therapy | 46 |
| Kileen 2004 | alcohol | USA | 50 mg | 12 weeks | psychotherapy (1 arm treatment as usual and no pills, not included in this analysis) | 37 |
| Kim 2001 | pathological gambling | USA | 50 to 250 mg max - average dose 187.5 mg in those who completed | 11 weeks (& 1 wk initial single blind placebo) |  | 49 |
| King 2006 | smoking | USA | 50 mg | 8 weeks 3 days | open label NRT for 1 month in decreasing dose & behavioural therapy | 44 |
| King 2012 | smoking | USA | 50 mg | 13 weeks | NRT & C.B. smoking cessation counselling | 42 |
| Kovanen 2016 | pathological gambling | Finland | 50 mg | 20 weeks | supportive counselling | 46 |
| Kranzler 2003 | Alcohol – mild to moderate alcohol dependence | USA | two arm study, 50 mg or 50 mg targeted reducing weekly to 0 | 8 weeks | brief coping skills counselling | 47 |
| Kranzler 2009 | alcohol | USA | two arm study 50 mg daily or 50 mg targeted, average = 39 mg / week | 12 weeks | brief coping skills counselling | 49 |
| Krishnan-Sarin 2003 | smoking | USA | 50 mg | 4 weeks | NRT, education & support | 39 |
| Krystal 2001 | alcohol | USA | 50 mg | 3 or 12 months | individual 12-step facilitation counselling | 49 |
| Latt 2002 | alcohol | Australia | 50 mg | 12 weeks | non-obligatory counselling | 45 |
| Lee 2001 | alcohol | Singapore | 50 mg | 12 weeks | 12 step total abstinence rehabilitation programme | 45 |
| Mann 2013 | alcohol | Germany | 50 mg | 12 weeks | 3 arms (acamprosate arm not included in this analysis) MM. If relapsed & hospital admission, re-randomised for MM +/- intensive CBT | 45 |
| Monterosso 2001 | alcohol | USA | 100 mg | 12 weeks, then some of naltrexone group crossed to placebo | psychosocial counselling | 46 |
| Mooney 2016 | smoking | USA | 50 mg | 7 weeks | bupropion or bupropion-placebo (2 arms) & brief behavioural counselling | 40 |
| Morgenstern 2012 | alcohol & homosexual men | USA | 100 mg | 12 weeks | +/- modified behavioural self-control therapy (4 arms factorial). All had brief behavioural compliance enhancement treatment | 40 |
| Morley 2006 | alcohol | Australia | 50 mg | 12 weeks | 3 arms (acamprosate arm not included in this analysis), manualised compliance therapy & medical care | 45 |
| Morris 2001 | alcohol | Australia | 50 mg | 12 weeks | education support group | 48 |
| Murphy 2014 | bipolar disorder with depression | USA | 50mg | 12 weeks | existing medication, stable for at least 2 weeks before enrolment | not stated - 18 to 65 |
| Niederhofer 2003 | alcohol in adolescents | Austria | 50 mg | 90 days |  | not stated - 16 to 19 |
| O'Malley 2006 | smoking | USA | 25 mg, 50 mg or 100 mg depending on randomised arm | 6 weeks | NRT & counselling | 46 |
| O'Malley 2007 | alcohol | USA | 50 mg | 12 weeks | group psychotherapy, C.B. coping skills therapy | 40 |
| O'Malley 2008 | alcohol | USA | 50 mg | 16 weeks | 3 arms (sertraline+NTX arm not included in this analysis), MM & supportive advice | 40 |
| O'Malley 2015 | alcohol in young adults | USA | 25 mg daily & 25 mg during craving | 8 weeks | personalised feedback session & brief counselling | 22 |
| Orexigen OT-101 2010 | obesity | USA | 50 mg | 16 weeks | bupropion SR (4 arms factorial) | not stated - 18 to 60 |
| Oslin 2005 | later life depression & alcohol | USA | 50 mg | 12 weeks | sertraline open label & supportive counselling | 63 |
| Oslin 2008 | alcohol | USA | 100 mg | 24 weeks | Supportive counselling +/- CBT or doctor only | 41 |
| Oslin 2015 | alcohol | USA | 50 mg | 12 weeks | MM | 49 |
| Papay 2014 | Parkinson’s diseases & impulse control disorder | USA | 50 or 100 mg if no response | 8 weeks |  | 61 |
| Peters 2015 | high grade glioma | USA | 4.5 mg | 16 weeks | standard chemoradiation (radiation and temozolomide) | 56 |
| Petrakis 2004 | schizophrenia & alcohol | USA | 50 mg | 12 weeks | standard neuroleptic medication, CBT drug relapse prevention strategies & skills training | 46 |
| Petrakis 2005 | axis 1 disorder & alcohol | USA | 50 mg | 12 weeks | disulfiram open label (4 arms factorial), intensive substance abuse treatment programme | 47 |
| Petrakis 2012 | PTSD & alcohol | USA | 50 mg | 12 weeks | paroxetine or desipramine (4 arms), standard medical care | 47 |
| Pettinati 2008a | cocaine & alcohol | USA | 100 mg | 11 weeks | disulfiram (4 arms factorial), CBT | 41 |
| Pettinati 2008b | cocaine & alcohol | USA | 150 mg | 12 weeks | CBT or psychosocial counselling | 39 |
| Pettinati 2010 | depression & alcohol | USA | 100 mg | 14 weeks | sertraline (4 arms factorial), CBT | 43 |
| Potenza 2017 | pathological gambling | USA | 50 mg | not stated | treatment as usual | 55 |
| Salloum 2011 | alcohol and major depressive disorder | USA | 50 mg | 24 weeks | fluoxetine open label | not stated - over 18 |
| Salloum 2014 | alcohol and bipolar disorder | USA | 50 mg | 12 weeks | valproate open label | 42 |
| Santos 2016 | Methamphetamine, alcohol & men who have sex with men | USA | 50 mg (1 tablet) during craving, mean weekly intake = 2.2 tablets | 8 weeks | brief substance use counselling | 43 |
| Schmitz 2001 | cocaine | USA | 50 mg | 12 weeks | relapse prevention therapy or drug counselling | 34 |
| Schmitz 2004 | cocaine & alcohol | USA | 50 mg | 12 weeks | relapse prevention therapy or drug counselling | 36 |
| Schmitz 2009 | cocaine & alcohol | USA | 100 mg | 12 weeks | CBT +/- CM with monetary rewards | 34 |
| Schmitz 2014 | cocaine | USA | 50 mg | 12 weeks | CBT and CM (2 arms not included in this analysis, modafinil or levodopa/carbidopa). | 42 |
| Seifrabiei 2008 | haematological malignancy | Iran | 3 mg | 21 weeks | routine treatment for haematological malignancies | 38 |
| Smith 2011 | Crohn’s disease | USA | 4.5 mg | 12 weeks | usual stable treatment for Crohn's disease | 43 |
| Smith 2013 | Crohn’s disease in children | USA | 0.1 mg/kg not to exceed 4.5 mg | 8 weeks | usual stable treatment for Crohn's disease | 12 |
| Spencer 2016 | Attention Deficit Disorder | USA | 100 mg | 6 weeks |  | 34 |
| Spencer 2018 | Attention Deficit Disorder | USA | 50 mg | 6 weeks | open label methylphenidate, subjects preselected by experiencing euphoria with test dose of methylphenidate | 25 |
| Taveira 2014 | olanzapine treated schizophrenia | USA | 50 mg | 12 weeks | stable dose of olanzapine | 45 |
| Tek 2014 | schizophrenia & obesity | USA | 25 mg | 8 weeks | stable anti-psychotic medication | 46 |
| Toll 2010a | smoking & weight loss | USA | 25 mg | 27 weeks | open label NRT, behavioural counselling | 44 |
| Toll 2010b | smoking and weight gain on stopping | USA | 25 mg | 11 weeks | open label varenicline, brief behavioural counselling based on CBT | 47 |
| Toneatto 2009 | pathological gambling & alcohol | Canada | up to 250 mg - mean dose 100 mg | 11 weeks | C.B. counselling | 40 |
| Wang 2018 | alcohol and HIV infection | USA | 50 mg | 120 days | anti-retroviral treatment where appropriate clinically | 48 |

abbreviations

AUD = alcohol use disorder

C.B. = cognitive behavioural

CBI = combined behavioural intervention

CBT = cognitive behaviour therapy

CM = contingency management

MM = medical management

NRT = nicotine replacement therapy, as prolonged release nicotine patches
