# Supplementary material for: Serious adverse events reported in placebo randomised controlled trials of oral naltrexone: a systematic review and meta-analysis
Source: BMC Med. 2019 Jan 15;17:10. doi: 10.1186/s12916-018-1242-0 (PMC6332608; doi:10.1186/s12916-018-1242-0)
Supplement: Supplementary file 5 — Table S3. Meta-analysis of adverse events data across trials (DOCX 24 kb) [file 12916_2018_1242_MOESM5_ESM.docx]

Additional file 5: Table S3:- Meta-analysis of adverse events data across trials

| **MedDRA Preferred-term Adverse event** | **No. of studies** | **RR** | **95% CI** | **I^2^** |
| --- | --- | --- | --- | --- |
| abdominal cramps | 1 | 3.7736 | [0.4365; 32.6222] |  |
| abdominal infection | 1 | 0.3456 | [0.0144; 8.3006] |  |
| abdominal pain | 7 | 1.4265 | [0.9987; 2.0375] | 25.70% |
| acid reflux | 1 | 0.3023 | [0.0131; 6.9945] |  |
| acute kidney injury | 1 | 1.037 | [0.1514; 7.1018] |  |
| agitation | 1 | 3.1101 | [0.1295; 74.7055] |  |
| agitation or anxiety | 1 | 0.9408 | [0.6936; 1.2761] |  |
| alanine aminotransferase increased | 1 | 0.8733 | [0.5038; 1.5137] |  |
| alkaline phosphatase increased | 1 | 0.6914 | [0.1202; 3.9773] |  |
| allergic reaction | 1 | 0.2073 | [0.0102; 4.2210] |  |
| allergic rhinitis | 1 | 1.037 | [0.0665; 16.1651] |  |
| alopecia | 1 | 0.5185 | [0.0484; 5.5533] |  |
| anemia | 1 | 0.7467 | [0.4635; 1.2028] |  |
| anorexia | 1 | 0.8296 | [0.3542; 1.9434] |  |
| anxiety | 7 | 1.1822 | [0.9086; 1.5380] | 0.00% |
| anxiety/tension | 2 | 0.9233 | [0.4745; 1.7963] | 0.00% |
| appetite changes | 1 | 0.5129 | [0.2408; 1.0922] |  |
| arthralgia | 1 | 0.6914 | [0.1202; 3.9773] |  |
| arthritis | 1 | 0.3456 | [0.0144; 8.3006] |  |
| aspartate aminotransferase increased | 1 | 1.1852 | [0.6426; 2.1860] |  |
| ataxia | 1 | 3.1101 | [0.1295; 74.7055] |  |
| back pain | 4 | 0.3439 | [0.0904; 1.3085] | 15.90% |
| baseline hemiparesis | 1 | 3.1101 | [0.1295; 74.7055] |  |
| blood bilirubin increased | 1 | 0.7778 | [0.2889; 2.0938] |  |
| blurred vision | 3 | 1.0558 | [0.5088; 2.1909] | 38.30% |
| buttock pain | 1 | 0.3456 | [0.0144; 8.3006] |  |
| cellulitis | 1 | 3.1101 | [0.1295; 74.7055] |  |
| cerebrospinal fluid leakage | 1 | 3.1101 | [0.1295; 74.7055] |  |
| change in libido | 3 | 0.8895 | [0.4797; 1.6495] | 0.00% |
| changes to vision | 1 | 4.0488 | [0.4623; 35.4587] |  |
| chest pain - cardiac | 1 | 3.1101 | [0.1295; 74.7055] |  |
| chest wall pain | 1 | 3.1101 | [0.1295; 74.7055] |  |
| chills | 1 | 3.1101 | [0.1295; 74.7055] |  |
| chipped tooth | 1 | 3.1101 | [0.1295; 74.7055] |  |
| cholecystitis | 1 | 1.037 | [0.0665; 16.1651] |  |
| cognitive disturbance | 1 | 3.1101 | [0.1295; 74.7055] |  |
| cold | 1 | 1.1962 | [0.5692; 2.5140] |  |
| conduction disorder | 1 | 3.1101 | [0.1295; 74.7055] |  |
| confusion | 1 | 3.1101 | [0.1295; 74.7055] |  |
| constipation | 4 | 1.1559 | [0.7110; 1.8792] | 34.20% |
| cough | 1 | 0.3457 | [0.0371; 3.2215] |  |
| cramps in feet | 1 | 3.1101 | [0.1295; 74.7055] |  |
| cramps in legs | 1 | 0.3456 | [0.0144; 8.3006] |  |
| creatinine increased | 1 | 1.037 | [0.3181; 3.3813] |  |
| **decreased appetite** | **7** | **1.443** | **[1.0917; 1.9073]** | **21.70%** |
| decreased grip strength in r-hand | 1 | 3.1101 | [0.1295; 74.7055] |  |
| dehydration | 1 | 0.3456 | [0.0144; 8.3006] |  |
| depression | 8 | 1.2099 | [0.8632; 1.6959] | 20.50% |
| dermatitis at wound site | 1 | 0.3456 | [0.0144; 8.3006] |  |
| diarrhea | 11 | 1.1477 | [0.8008; 1.6448] | 68.10% |
| difficulty sleeping | 1 | 2.8302 | [0.3043; 26.3188] |  |
| diverticulitis | 1 | 0.3456 | [0.0144; 8.3006] |  |
| diverticulitis baseline | 1 | 0.3456 | [0.0144; 8.3006] |  |
| **dizziness** | **13** | **1.4512** | **[1.1501; 1.8312]** | **0.00%** |
| dizzyness | 1 | 2.7209 | [0.1176; 62.9503] |  |
| drowsiness | 2 | 1.2439 | [0.5009; 3.0887] | 0.00% |
| dry mouth | 6 | 1.4304 | [0.6227; 3.2856] | 17.40% |
| dry skin | 1 | 1.037 | [0.1514; 7.1018] |  |
| dysgeusia | 1 | 0.7778 | [0.1825; 3.3142] |  |
| dyspepsia | 1 | 0.5185 | [0.0484; 5.5533] |  |
| dysphagia | 1 | 0.3456 | [0.0144; 8.3006] |  |
| dysphasia | 1 | 3.1101 | [0.1295; 74.7055] |  |
| dyspnea | 1 | 11.4037 | [0.6459; 201.3366] |  |
| edema cerebral | 1 | 0.3456 | [0.0144; 8.3006] |  |
| edema face | 1 | 0.3456 | [0.0144; 8.3006] |  |
| edema limbs | 1 | 2.5926 | [0.5252; 12.7982] |  |
| epistaxis | 1 | 0.6914 | [0.1202; 3.9773] |  |
| fall | 1 | 5.1835 | [0.2546; 105.5254] |  |
| fatigue | 10 | 1.2405 | [0.9788; 1.5721] | 23.20% |
| fatigue or low energy | 1 | 2.2013 | [0.6022; 8.0459] |  |
| fecal incontinence | 1 | 0.3456 | [0.0144; 8.3006] |  |
| fever | 1 | 1.5556 | [0.2704; 8.9490] |  |
| flatulence | 1 | 1.8095 | [0.1780; 18.3920] |  |
| flu | 1 | 0.3456 | [0.0144; 8.3006] |  |
| flu-like symptoms | 1 | 1.0132 | [0.3781; 2.7148] |  |
| flushed or warm | 1 | 1.2477 | [0.7675; 2.0284] |  |
| gait disturbance | 1 | 0.3456 | [0.0144; 8.3006] |  |
| gastritis | 1 | 0.3456 | [0.0144; 8.3006] |  |
| gastroenteritis | 2 | 2.3308 | [0.4589; 11.8381] | 0.00% |
| gastroesophageal reflux disease | 1 | 5.1835 | [0.2546; 105.5254] |  |
| generalized muscle weakness | 1 | 2.0741 | [0.1937; 22.2132] |  |
| gi bug | 1 | 1.0367 | [0.0209; 51.3295] |  |
| h-pylori | 1 | 3.1101 | [0.1295; 74.7055] |  |
| headache | 14 | 1.0421 | [0.8563; 1.2681] | 24.70% |
| hematoma | 1 | 0.3456 | [0.0144; 8.3006] |  |
| hemorrhoids | 1 | 3.1101 | [0.1295; 74.7055] |  |
| hoarseness | 1 | 2.5926 | [0.5252; 12.7982] |  |
| hyperglycemia | 1 | 1.1852 | [0.8412; 1.6698] |  |
| hyperkalemia | 1 | 1.5556 | [0.2704; 8.9490] |  |
| hypernatremia | 1 | 1.7284 | [0.4341; 6.8818] |  |
| hypersomnia | 1 | 3.1101 | [0.1295; 74.7055] |  |
| hypertension | 1 | 1.5556 | [0.2704; 8.9490] |  |
| hypoalbuminemia | 1 | 0.7778 | [0.3568; 1.6954] |  |
| hypocalcemia | 1 | 1.6593 | [0.8272; 3.3282] |  |
| hypoglycemia | 1 | 0.4444 | [0.1211; 1.6305] |  |
| hypokalemia | 1 | 1.5556 | [0.7667; 3.1560] |  |
| hyponatremia | 1 | 0.7407 | [0.3605; 1.5222] |  |
| hypotension | 1 | 0.2073 | [0.0102; 4.2210] |  |
| ileal obstruction | 1 | 0.3456 | [0.0144; 8.3006] |  |
| increased appetite | 3 | 1.1395 | [0.8345; 1.5558] | 0.00% |
| increased bowel frequency | 2 | 0.7345 | [0.3729; 1.4467] | 0.00% |
| increased erections | 1 | 1.518 | [0.8249; 2.7936] |  |
| increased irritability | 1 | 0.6748 | [0.1977; 2.3035] |  |
| increased sexual desire | 1 | 1.1511 | [0.7677; 1.7261] |  |
| insomnia | 10 | 0.9772 | [0.7321; 1.3043] | 19.10% |
| intracranial hemorrhage | 1 | 0.3456 | [0.0144; 8.3006] |  |
| irregular menses | 1 | 1.0984 | [0.2871; 4.2022] |  |
| irritability | 4 | 2.0157 | [0.3585; 11.3332] | 13.60% |
| itchiness | 1 | 0.7292 | [0.4002; 1.3284] |  |
| itching | 2 | 1.0555 | [0.6616; 1.6838] | 14.70% |
| joint and muscle pain | 1 | 0.3456 | [0.0144; 8.3006] |  |
| joint or muscle pain | 1 | 0.3145 | [0.0338; 2.9243] |  |
| joint pain | 1 | 1.8095 | [0.1780; 18.3920] |  |
| lightheaded or dizzy | 1 | 1.3072 | [0.7973; 2.1431] |  |
| lipase increased | 1 | 0.3456 | [0.0144; 8.3006] |  |
| loose stool | 1 | 0.5061 | [0.0953; 2.6877] |  |
| lyme's dx | 1 | 3.1101 | [0.1295; 74.7055] |  |
| lymphocyte count decreased | 1 | 0.8815 | [0.5201; 1.4939] |  |
| macular errythematous | 1 | 3.1101 | [0.1295; 74.7055] |  |
| memory impairment | 1 | 3.1111 | [0.3338; 28.9933] |  |
| mucositis oral | 1 | 0.5185 | [0.1365; 1.9695] |  |
| muscle aches | 1 | 0.7591 | [0.1753; 3.2869] |  |
| muscle twitch | 1 | 4.7196 | [0.2322; 95.9324] |  |
| muscle weakness | 1 | 0.7515 | [0.3486; 1.6204] |  |
| muscle weakness left-sided | 1 | 5.1835 | [0.2546; 105.5254] |  |
| muscle weakness lower limb | 1 | 1.037 | [0.0665; 16.1651] |  |
| muscle weakness right-sided | 1 | 5.1835 | [0.2546; 105.5254] |  |
| myalgia | 1 | 1.037 | [0.0665; 16.1651] |  |
| myositis | 1 | 3.1101 | [0.1295; 74.7055] |  |
| nasal congestion | 1 | 3.1101 | [0.1295; 74.7055] |  |
| nasopharyngitis | 2 | 1.1746 | [0.3220; 4.2854] | 0.00% |
| **nausea** | **18** | **1.5885** | **[1.3718; 1.8394]** | **22.20%** |
| nausea or upset stomach | 1 | 1.5723 | [0.7573; 3.2643] |  |
| neck pain | 1 | 3.1101 | [0.1295; 74.7055] |  |
| nervousness/anxiety | 2 | 0.9301 | [0.5740; 1.5071] | 0.00% |
| neutrophil count decreased | 1 | 0.5185 | [0.2556; 1.0520] |  |
| nightmares | 1 | 2.7209 | [0.1176; 62.9503] |  |
| non-cardiac chest pain | 1 | 3.1101 | [0.1295; 74.7055] |  |
| otitis external | 1 | 0.3456 | [0.0144; 8.3006] |  |
| pain | 2 | 0.4352 | [0.0646; 2.9335] | 0.00% |
| pain in extremity | 1 | 0.5185 | [0.0484; 5.5533] |  |
| papulopustular rash | 1 | 3.1101 | [0.1295; 74.7055] |  |
| paraesthesia | 2 | 0.4128 | [0.0918; 1.8569] | 0.00% |
| paresthesia | 1 | 5.1835 | [0.2546; 105.5254] |  |
| peripheral sensory neuropathy | 1 | 3.1101 | [0.1295; 74.7055] |  |
| phosphorus elevated | 1 | 0.3456 | [0.0144; 8.3006] |  |
| platelet count decreased | 1 | 0.9701 | [0.6895; 1.3651] |  |
| pneumonitis | 1 | 3.1101 | [0.1295; 74.7055] |  |
| postnasal drip | 1 | 0.8889 | [0.3191; 2.4758] |  |
| premature ejaculation | 1 | 0.5 | [0.1608; 1.5547] |  |
| proteinuria | 1 | 0.6222 | [0.1563; 2.4774] |  |
| pruritus | 2 | 1.0028 | [0.0159; 63.0967] | 81.50% |
| pyramidal tract syndrome | 1 | 0.3456 | [0.0144; 8.3006] |  |
| radiation recall reaction | 1 | 3.1101 | [0.1295; 74.7055] |  |
| rapid heart beat | 1 | 0.9256 | [0.5228; 1.6386] |  |
| rash | 3 | 0.7882 | [0.2918; 2.1293] | 65.90% |
| rash acneiform | 1 | 0.5185 | [0.0484; 5.5533] |  |
| rash maculo-papular | 1 | 1.3827 | [0.3245; 5.8919] |  |
| renal calculi | 1 | 3.1101 | [0.1295; 74.7055] |  |
| sedation | 1 | 1.3496 | [0.3117; 5.8434] |  |
| seizure | 1 | 2.0741 | [0.5461; 7.8778] |  |
| shingles | 1 | 1.037 | [0.0665; 16.1651] |  |
| sinus bradycardia | 1 | 0.3456 | [0.0144; 8.3006] |  |
| sinusitis | 1 | 0.2073 | [0.0102; 4.2210] |  |
| skin rash | 2 | 0.6739 | [0.1603; 2.8340] | 11.30% |
| sleep disturbance | 1 | 2.9731 | [0.1224; 72.1956] |  |
| **sleepiness** | **5** | **1.452** | **[1.0697; 1.9709]** | **2.00%** |
| somnolence | 6 | 1.1842 | [0.8677; 1.6160] | 60.50% |
| sore throat | 1 | 3.1101 | [0.1295; 74.7055] |  |
| stomach pain | 1 | 0.6748 | [0.1977; 2.3035] |  |
| stomach virus | 1 | 7.0848 | [0.3717; 135.0283] |  |
| **sweating** | **3** | **1.8932** | **[1.2471; 2.8742]** | **0.00%** |
| thromboembolic event | 1 | 5.1852 | [0.6260; 42.9504] |  |
| tiredness | 2 | 1.1326 | [0.7475; 1.7162] | 37.40% |
| toothache | 1 | 0.6073 | [0.1500; 2.4590] |  |
| tremor | 1 | 2.0741 | [0.3961; 10.8614] |  |
| upper respiratory infection | 1 | 4.1481 | [0.4788; 35.9399] |  |
| urinary incontinence | 1 | 1.037 | [0.0665; 16.1651] |  |
| urinary tract infection | 1 | 1.037 | [0.1514; 7.1018] |  |
| ventricular tachycardia | 1 | 0.3456 | [0.0144; 8.3006] |  |
| visual field cut in both lower | 1 | 0.3456 | [0.0144; 8.3006] |  |
| vivid dreams | 4 | 1.0536 | [0.6418; 1.7296] | 38.50% |
| **vomiting** | **13** | **1.9107** | **[1.5090; 2.4192]** | **0.00%** |
| weight gain | 1 | 3.1101 | [0.1295; 74.7055] |  |
| weight loss | 1 | 2.5926 | [0.5252; 12.7982] |  |
| white blood cell decreased | 1 | 0.8116 | [0.4969; 1.3256] |  |
| wound complication | 1 | 0.2073 | [0.0102; 4.2210] |  |
| wound infection | 1 | 0.3456 | [0.0144; 8.3006] |  |
| **Total number of events** | **Naltrexone arm = 3938; Placebo arm = 3079** | | | |
