# Supplementary figures and images for: Serious adverse events reported in placebo randomised controlled trials of oral naltrexone: a systematic review and meta-analysis
Source: BMC Med. 2019 Jan 15;17:10. doi: 10.1186/s12916-018-1242-0 (PMC6332608; doi:10.1186/s12916-018-1242-0)

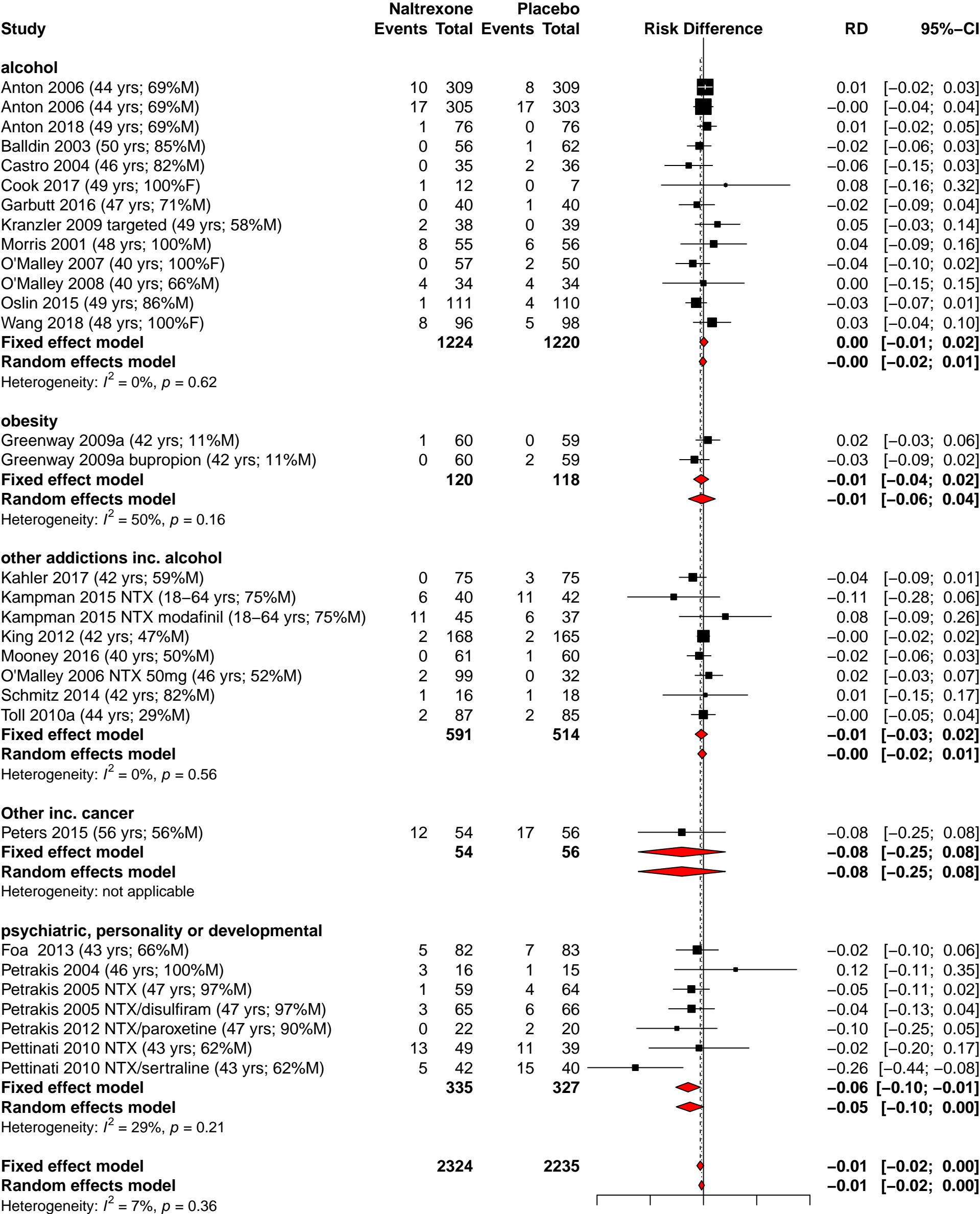

Supplement: Supplementary file 6 — Figure S1. Subgroup analysis of SAEs by disease type (PDF 13 kb) [file 12916_2018_1242_MOESM6_ESM.pdf]

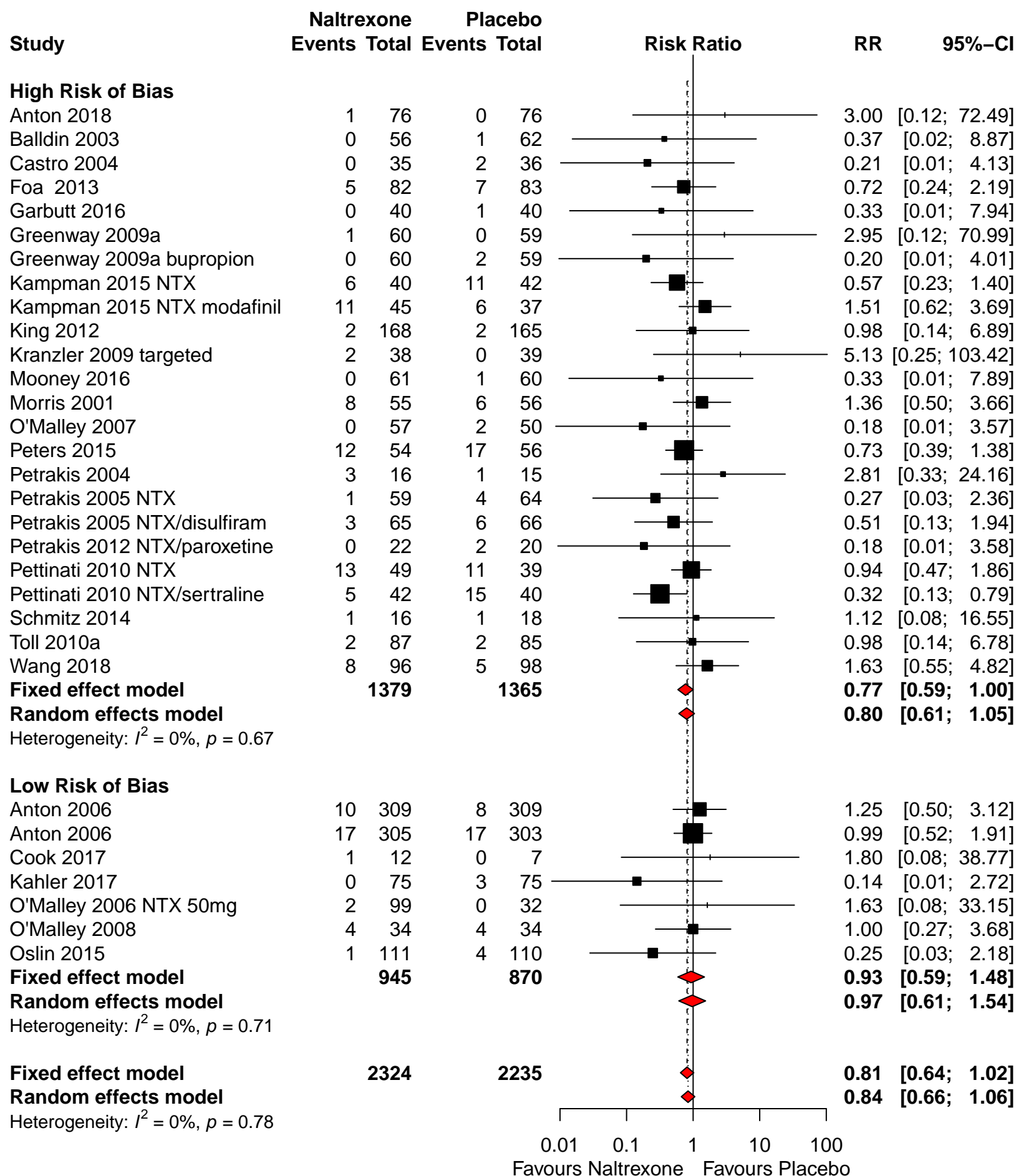

Supplement: Supplementary file 7 — Figure S2. Forest plot of SAEs by risk of bias (PDF 13 kb) [file 12916_2018_1242_MOESM7_ESM.pdf]
